# Is Having Sex with Other Men a Risk Factor for Transfusion-Transmissible Infections in Male Blood Donors in Western Countries? A Systematic Review

**DOI:** 10.1371/journal.pone.0122523

**Published:** 2015-04-15

**Authors:** Emmy De Buck, Tessa Dieltjens, Veerle Compernolle, Philippe Vandekerckhove

**Affiliations:** 1 Centre for Evidence-Based Practice, Belgian Red Cross-Flanders, Mechelen, Belgium; 2 Blood Service, Belgian Red Cross-Flanders, Mechelen, Belgium; 3 Faculty of Medicine and Health Sciences, University of Ghent, Ghent, Belgium; 4 Belgian Red Cross-Flanders, Mechelen, Belgium; 5 Department of Public Health and Primary Care, Faculty of Medicine, Catholic University of Leuven, Leuven, Belgium; 6 Faculty of Medicine, University of Ghent, Ghent, Belgium; Rollins School of Public Health, Emory University, UNITED STATES

## Abstract

**Background:**

Although increased prevalence of transfusion transmissible infections (TTI) among “men who have sex with men” (MSM) has been well documented, the exclusion of MSM as blood donors is contested. The aim of this systematic review is to find studies that describe the risk of TTI in MSM blood donors.

**Methods:**

We searched MEDLINE, Embase, The Cochrane Central Register of Controlled Trials, Cinahl, and Web of Science, and used GRADE for determining evidence quality. We included studies comparing MSM and non-MSM blood donors (or people eligible to give blood), living in areas most relevant for our Blood Service.

**Results:**

Out of 18 987 articles, 14 observational studies were included. Two studies directly compared MSM with non-MSM donors showing that MSM donors have a statistically significant higher risk of HIV-1 infections. In one of these studies it was shown that this was related to recent (< 12 months) MSM contact. In two additional studies no evidence was shown in favour of a certain deferral period for MSM. Ten studies, applying permanent deferral for MSM, compared infected versus non-infected donors. One study found that MSM is a statistically significant risk factor for HIV-1 infection in blood donors. For other TTI such as HBV or HCV, an increased risk of infection could not be demonstrated, because the precision of the results was affected by the low numbers of donors with MSM as risk factor, or because of risk of bias in the included studies. All studies included low level evidence, because of risk of bias and imprecision of the results.

**Conclusions:**

High-quality studies investigating the risk of TTI in MSM who donate blood are scarce. The available evidence suggests a link between MSM blood donors and HIV-1 infection, but is too limited to be able to unambiguously/clearly recommend a certain deferral policy.

## Background

In order to guarantee a safe blood supply blood donors must meet certain eligibility requirements. Eligibility is determined through the donor history questionnaire and interview, assessing risk factors for transfusion transmissible infections (TTI) such as tattooing, injecting drugs, and sexual behaviour such as “men who have sex with other men” (MSM). MSM have the highest prevalence of human immunodeficiency virus (HIV) in the Western world, with rising incidence in many countries. Other sexually transmitted diseases (STD) which are also transmissible via blood or transfusion, are hepatitis B and hepatitis C, with a high prevalence of hepatitis B virus (HBV) infection in MSM and a rising incidence of hepatitis C virus (HCV) infection in HIV-infected MSM. In addition, recent increases of syphilis in MSM have been documented, and MSM are also more often diagnosed with other STDs which are transfusion transmissible, such as chlamydia [1,2]. Whether that in itself justifies excluding MSM from donating blood has been the subject of public dispute, the question being whether the risk of infection is high enough to support exclusion, and if so for how long [3,4]? One side brands the measures as discriminatory against gay men [5]. The other side argues that exclusion is based on MSM behaviour rather than sexual orientation, that the epidemiology of TTI is clear, and that the right of the patient to receive safe blood takes precedence over the wish of a particular donor group to be allowed to donate blood [6]. Measures differ widely between countries, ranging from lifelong to a five or one year exclusion, or even no exclusion at all [7].

This article is the first systematic review on this topic, and uses the principles of Evidence-Based Medicine at its most rigorous level, by applying the methodology of the Cochrane Collaboration [8]. The aim was to find studies that describe the relationship between MSM and safe blood donation, not the prevalence of TTI in MSM in general for which systematic reviews already exist [1]. The following ‘PICO’ question was developed: For male blood donors (Population) is having sex with other men (Intervention) a risk factor for TTI (Outcome) compared to not having sex with other men (Comparison) in Western countries?

## Methods

We followed the PRISMA statement for the reporting of this systematic review [9]. No protocol for this systematic review existed or was published beforehand.

### Selection criteria

We used the following inclusion and exclusion criteria for the selection of articles:

Population: Inclusion: blood donors (or people eligible to give blood), living in areas most relevant for our Blood Service: Northern, Western, and Southern Europe (Albania, Andorra, Austria, Belgium, Bosnia and Herzegovina, Croatia, Denmark, Estonia, Finland, France, Germany, Gibraltar, Greece, Iceland, Italy, Ireland, Kosovo, Latvia, Liechtenstein, Lithuania, Luxembourg, Macedonia, Malta, Monaco, Montenegro, the Netherlands, Norway, Portugal, San Marino, Serbia, Slovenia, Spain, Sweden, Switzerland, Vatican City), USA, Canada, Australia, New-Zealand; Exclusion: a population containing blood donors, but not exclusively consisting of blood donors.

Intervention/Risk factor: Inclusion: men having sex with other men after 1977.

Comparison: Inclusion: men not having sex with other men.

Outcome: Inclusion: markers of transfusion-transmissible infections from the following pathogenic micro-organisms in the donor blood (which are sexually transmitted and also transfusion transmissible): HIV, HBV, HCV, *Chlamydia*, and *Treponema pallidum* (causing syphilis).

Study design: Inclusion: Intervention studies: randomized controlled trials, controlled clinical trials, before- and after studies; Observational studies: cohort studies, case-control studies, cross-sectional studies (surveys); these study types are included independently of the potential risk of bias (however, risk of bias will transparently be reported); Exclusion: non-controlled studies, case reports, case series, letters, comments, opinion pieces, narrative reviews, modelling studies.

No language criteria were used.

### Search strategy and study selection

There are three types of studies that can potentially answer our question: (1) “Type 1 studies” comparing the incidence of TTI in blood products of MSM and non-MSM donors, (2) “Type 2 studies” comparing two types of deferral strategies for MSM donors, (3) “Type 3 studies” comparing infected blood donors (cases) versus non-infected blood donors (controls), identifying risk factors (e.g. MSM) of both groups (case-control studies).

Search strategies were composed to retrieve the three study types. The following databases were searched from their date of inception to 26 March 2014: MEDLINE (using the PubMed interface), Embase (using the Embase.com interface), The Cochrane Central Register of Controlled Trials, Cinahl, and Web of Science. Full details of the search strategies are given in S1 File. Study selection was performed in parallel by two independent reviewers (EDB, TD). Titles and abstracts of the studies identified by the search were scanned. When a relevant article was found, full text articles were retrieved. Studies that did not meet the selection criteria were excluded. The citation and reference lists of included studies were searched, and the first 20 related items in PubMed were scanned for other potentially relevant studies. Any discrepancies among the two reviewers were resolved by consensus.

### Data collection

Data concerning study design, study population, outcome measures (markers of transfusion-transmissible infections expressed as risk ratio, odds ratio or incidence rate ratio), and study quality were extracted independently by two reviewers (EDB, TD). In the event that data were lacking, authors were contacted to obtain more detailed information. No statistical methods were used to pool the data because of heterogeneity of the studies.

### Quality of evidence

The GRADE approach was used to assess the overall quality of evidence included in this review and the online available Guideline Development Tool (GDT), developed by the GRADE Working Group, was used to develop the GRADE table, including quality assessment and a summary of findings (http://www.guidelinedevelopment.org/). This table was adapted manually in case our data were not compatible with the template. A level of evidence was assigned for each outcome, which were all rated as critical outcomes. GRADE downgrades the level of evidence because of risk of bias (limitations in study design) of the included studies, inconsistency between results of different studies (due to differences in populations, interventions or outcomes), indirectness (of population, intervention or outcome), imprecision and publication bias. All studies included in this systematic review were observational studies, which results in an initial “low level of evidence” according to the GRADE approach. For none of the observational studies included there were reasons to upgrade the level of evidence. Risk of bias in the individual studies was analysed at the study level by evaluating the presence of eligibility criteria, adequate control of confounding, follow-up and correct measurement of exposure and outcome, as detailed in the Help section of the GDT [10].

## Results

### Study selection and study characteristics

**Fig 1:**
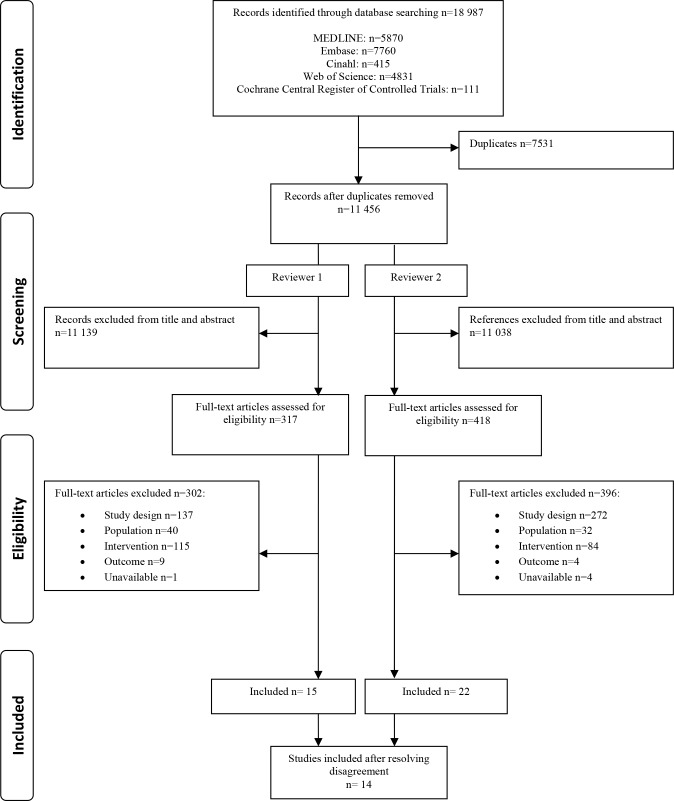
PRISMA flowchart of identification and selection of studies.

Fig 1 provides a flowchart of the identification and selection of studies. The searches yielded 18,987 references. After removing duplicates and screening relevant titles and abstracts, 317 references were selected by reviewer 1 and 418 references by reviewer 2 for which the full text was evaluated. In S1 Table an overview is given of all studies excluded by at least one of the reviewers, with the reason for exclusion. The major reason for exclusion was study design (56%) as many references were opinion pieces, letters, comments or uncontrolled studies. After resolving disagreements among the two reviewers, we retained 14 observational studies (no references of a higher study design were found), which are described below and in detail in Table 1.

**Table 1:** Characteristics of included studies.

**Author, Year, Country**	**Study design**	**Population**	**Comparison/Risk factor**	**Remarks**
Allison, 2012, United States [11]	Observational study: case-control	469 true HCV positive volunteer blood donors; 217 false HCV positive volunteer blood donors; 52 indeterminate HCV positive volunteer bloods	Risk factor: Men who have sex with other men	All subjects had positive anti-HCV findings by EIA. Donors with positive results of RIBA are considered infected with HCV
Alpaugh 1985, United States [12]	Observational study: cohort study	68 homosexuals, of which 52 were eligible donors on the basis of their medical history, and 209 random blood donors	Homosexuals versus non-homosexuals	Risk factor analysis was done only with the homosexuals who were eligible donors on the basis of their medical history. HBV was measured using anti-HBc testing (Corzyme EIA test) and HBsAg testing (Ayszyme II test).
Busch, 1994, United States [13]	Observational study: case-control	Blood donors: 146 HIV-1 infected cases and 151 uninfected controls enrolled; 129 and 131 replied	Risk factor: Males having sexual contact with males	HIV-1 positive was defined as positive in EIA, protein immunoblot and RIP
Christensen, 2001, Denmark [14]	Observational study: case-control	Registered blood donors (in 1997): 44 confirmed anti-HBc positive cases; 585 anti-HBc negative controls	Risk factor: Homosexual/bisexual male contact	
Kaldor, 1992, Australia [15]	Observational study: case-control	Blood donors in Sydney: 220 HCV infected cases (142 male, 78 female); 210 HCV uninfected controls (126 male, 84 female)	Risk factor: Homosexual (male)	HCV positive was defined as positive in two ELISAs, RIBA-2 and PCR.
Moore, 1997, United Kingdom [16]	Observational study: case-control	Blood donors (in 1993): 35 confirmed anti-HBc positive donors; 50 anti-HBc negative donors, of which 11 attended for interview	Risk factor: Men with homosexual partners	A group of confirmed anti-HBc positive cases excluded before donation for other reasons, were not included in our analysis.
Murphy, 2000, United States [17]	Observational study: case-control	Blood donors in 5 US Centres: 758 HCV infected cases; 1039 HCV uninfected controls	Risk factor: Men who have sex with (at least one) other men	HCV positive was defined as positive in ELISA assay and RIBA.
Neal, 1994, United Kingdom [18]	Observational study: case-control	Blood donors in Trent: 74 HCV infected cases (142 male, 78 female); 150 HCV uninfected controls (126 male, 84 female)	Risk factor: Homosexual (male)	HCV positive was defined as positive in ELISA assay and RIBA-2.
Sanchez 2005, United States [19]	Observational study:cross-sectional (survey)	25168 male blood donors	MSM vs. non-MSM donors; different periods since last MSM contact (self-reported in anonymous mail survey)	Outcome: Risk of TTI (reactive screening test result for HBc antigen, syphilis, HIV, human T-lymphotropic virus, HCV, HBs antigen, and ALAT)
Seed, 2010, Australia [20]	Observational study: before and after study	4,025,571 donations in 5-year period preceding implementation of 12-month MSM deferral and 4,964,628 donations in 5-year period post-implementation	HIV-positive male donors before and after implementation of 12-month deferral for MSM donors	
Serfaty, 1993, France [21]	Observational study: case-control	Blood donors with no history of transfusion or IDU: 35 HCV infected cases; 35 HCV uninfected controls	Risk factor: Homosexual	HCV positive was defined as positive in ELISA assay and RIBA-2.
Suligoi, 2013, Italy [22]	Observational study: before and after study	868,391 blood donors in 1999, 1,794,436 blood donors in 2009 and 1,840,464 blood donors in 2010	HIV-positive donors reporting MSM in a permanent deferral period (1999) versus a period of individual risk assessment (2009 and 2010)	
Tullen, 1993, Switzerland [23]	Observational study: case-control	Blood donors: 68 HCV infected cases; 103 HCV uninfected controls	Risk factor: Homosexual	HCV positive was defined as positive in ELISA assay and RT-PCR. Controls had high ALAT levels.
Van der Poel, 1991, the Netherlands [24]	Observational study: case-control	Blood donors in Amsterdam: 12 HCV infected cases; 113 HCV uninfected controls	Risk factor: Homosexual (male)	HCV positive was defined as positive in ELISA assay and 4-RIBA and PCR. All subjects had positive anti-HCV findings by ELISA.

ALAT: alanine aminotransferase; EIA: enzyme immunoassay; ELISA: enzyme-linked immunosorbent assay; HBc: hepatitis B core antigen; HBs: hepatitis B surface antigen; HBV: hepatitis B virus; HCV: hepatitis C virus; HIV: human immunodeficiency virus; IDU: intravenous drug use; MSM: men who have sex with men; RIBA: recombinant immunoblot assay; RNA: ribonucleic acid; RIP: radioimmunoprecipitation; (RT)-PCR: (reverse transcriptase) polymerase chain reaction; TTI: transfusion transmissible infections

#### Type 1 studies: comparison of TTI in MSM vs non-MSM donors

In two studies comparing MSM and non-MSM donors, TTI were searched for in respectively 25,168 male blood donors with different time intervals since the last MSM contact (self-reported in an anonymous mail survey) versus control donors, and in 52 MSM-donors who were eligible as blood donors versus 209 non-MSM blood donors [12,19].

#### Type 2 studies: comparison of TTI between different deferral strategies for MSM donors

Two studies compared different deferral strategies [20,22]. In the first study blood samples from donors, including MSM, were examined for HIV prevalence during the five years preceding and the five years following a change from a permanent/five-year deferral versus a one-year deferral for MSM [20]. The second study compared permanent deferral for MSM donors, with individual risk assessment of sexual behaviours [22].

#### Type 3 studies: comparison of infected vs non-infected blood donors for risk factors

10 case-control studies tried to identify risk factors for TTI in blood donors. The cases included blood donors with a viral infection with HIV-1 [13], HBV [14,16], or HCV [11,15,17,18,21,23,24], and the controls were donors who did not have this particular infection. Studies only looking at blood donors with infections were not included. Two of the 10 studies, where “homosexuality” was measured as a risk factor, did not explicitly mention if this concerned only men, but we assumed this was the case [21,23]. The majority of studies did not explicitly mention which donor selection strategy was in place with regards to MSM at the time of the study, however from other references we know that all studies had a policy of lifelong exclusion at the time of the study [7,20].

### Synthesis of findings

An overview of the synthesis of findings of all included studies can be found in Table 2. Since we were not able to pool any studies, a narrative overview of the results of the individual studies is given below

**Table 2:** Quality assessment and summary of findings according to the GRADE approach.

**Type 1 studies: comparison of TTI in MSM vs non-MSM donors**												
**Quality assessment**							**№ of patients**		**Effect**		**Quality**	**Importance**
**№ of studies**	**Study design**	**Risk of bias**	**Inconsistency**	**Indirectness**	**Imprecision**	**Other considerations**	**MSM**	**No MSM**	**Relative (95% CI)**	**Absolute (95% CI)**		
Presence of antibody to Hepatitis B core antigen (Alpaugh, 1985) [12]												
1	observational studies	serious ^1^	not serious	not serious	serious ^15^	none	16/52 (30.8%)	7/209 (3.3%)	**RR 9.19** (3.99 to 21.17) ^16^	274 more per 1000 (from 100 more to 676 more)	⨁◯◯◯ VERY LOW	CRITICAL
Presence of Hepatitis B surface antigen (Alpaugh, 1985) [12]												
1	observational studies	serious ^1^	not serious	not serious	serious ^15^	none	0/52 (0.0%)	0/209 (0.0%)	not estimable	not estimable	⨁◯◯◯ VERY LOW	CRITICAL
Presence of antibody to human T cell lymphotropic virus type III- antigen (= HIV-1), Initial reactive (Alpaugh, 1985) [12]												
1	observational studies	serious ^1^	not serious	not serious	serious ^15^	none	7/52 (13.5%)	4/205 (2.0%)	**RR 6.9** (2.1 to 22.68) ^16^	115 more per 1000 (from 21 more to 423 more)	⨁◯◯◯ VERY LOW	CRITICAL
Presence of antibody to human T cell lymphotropic virus type III- antigen (= HIV-1), Repeatable reactive (Alpaugh, 1985) [12]												
1	observational studies	serious ^1^	not serious	not serious	serious ^15^	none	7/52 (13.5%)	4/205 (2.0%)	**RR 27.6** (3.47 to 219.37) ^16^	519 more per 1000 (from 48 more to 4261 more)	⨁◯◯◯ VERY LOW	CRITICAL
Risk of transfusion transmissible viral infections (reactive screening test result) (Sanchez, 2005) [19]												
1	observational studies	serious ^2^	not serious	not serious	serious ^15^	none	MSM before 1977: 8/289 ^18^ (2.8%)	423/24879 (1.7%)	**aOR 1.7** (0.8 to 3.7)	12 more per 1000 (from 3 fewer to 43 more)	⨁◯◯◯ VERY LOW	CRITICAL
Risk of transfusion transmissible viral infections (reactive screening test result) (Sanchez, 2005) [19]												
1	observational studies	serious ^2^	not serious	not serious	serious ^15^	none	MSM after 1977: 17/280 ^18^ (6%)	423/24879 (1.7%)	**aOR 2.5** (1.4 to 4.5)	24 more per 1000 (from 7 more to 55 more)	⨁◯◯◯ VERY LOW	CRITICAL
Risk of transfusion transmissible viral infections (reactive screening test result) (Sanchez, 2005) [19]												
1	observational studies	serious ^2^	not serious	not serious	serious ^15^	none	MSM ≤ 12 months: 8.3% ^19^	423/24879 (1.7%)	**aOR 2.9** (1.5 to 5.8)	not estimable	⨁◯◯◯ VERY LOW	CRITICAL
Risk of transfusion transmissible viral infections (reactive screening test result) (Sanchez, 2005) [19]												
1	observational studies	serious ^2^	not serious	not serious	serious ^15^	none	MSM > 12 months-5 years: 6/54 ^18^ (11.1%)	423/24879 (1.7%)	**aOR 4.3** (0.9 to 21.7)	52 more per 1000 (from 2 fewer to 256 more)	⨁◯◯◯ VERY LOW	CRITICAL
Risk of transfusion transmissible viral infections (reactive screening test result) (Sanchez, 2005) [19]												
1	observational studies	serious ^2^	not serious	not serious	serious ^15^	none	MSM > 5 years: 2.3% ^19^	423/24879 (1.7%)	**aOR 1** (0.5 to 2.2)	not estimable	⨁◯◯◯ VERY LOW	CRITICAL
**Type 2 studies: comparison of TTI between different deferral strategies for MSM donors**												
**Quality assessment**							**№ of patients**		**Effect**		**Quality**	**Importance**
**№ of studies**	**Study design**	**Risk of bias**	**Inconsistency**	**Indirectness**	**Imprecision**	**Other considerations**	**Deferral strategy 1**	**Deferral strategy 2**	**Relative (95% CI)**	**Absolute (95% CI)**		
HIV-positive male donors (Seed, 2010) [20]												
1	observational study	serious ^3^	not serious	not serious	serious ^15^	none	1 year deferral: 13/2592473 (0.0%)	≥ 5 year deferral: 16/2106350 (0.0%)	**Incidence Rate Ratio 0.66** (0.32 to 1.37)	0 fewer per 1000 patient(s) per years (from 0 fewer to 0 fewer)	⨁◯◯◯ VERY LOW	CRITICAL
HIV-positive male donors reporting MSM (Suligoi, 2013) [22]												
1	observational study	serious ^4^	not serious	not serious	serious ^15^	none	Individual risk assessment: 2540362 (number of male donations)	Permanent deferral: data lacking on number of male donations	**RR 2.8** ^17^	not estimable	⨁◯◯◯ VERY LOW	CRITICAL
**Type 3 studies: comparison of infected vs non-infected blood donors for risk factors**												
**Quality assessment**							**№ of patients**		**Effect**		**Quality**	**Importance**
**№ of studies**	**Study design**	**Risk of bias**	**Inconsistency**	**Indirectness**	**Imprecision**	**Other considerations**	**MSM**	**no MSM**	**Relative (95% CI)**	**Absolute (95% CI)**		
Risk of HIV (Busch, 1994) [13]												
1	observational study	serious ^5^	not serious	not serious	serious ^15^	none	53/129 (41.1%)	2/131 (1.5%)	**OR 45** (10.66 to 189.84) ^16^	396 more per 1000 (from 127 more to 731 more)	⨁◯◯◯ VERY LOW	CRITICAL
Risk of HBV (Christensen, 2001) [14]												
1	observational study	serious ^6^	not serious	not serious	serious ^15^	none	1/37 (2.7%)	3/553 (0.5%)	**aOR 5.44** (p = 0.259) ^20^	23 more per 1000 (from 3 fewer to 210 more)	⨁◯◯◯ VERY LOW	CRITICAL
Risk of HBV (Moore, 1997) [16]												
1	observational study	serious ^7^	not serious	not serious	serious ^15^	none	2/35 (5.7%)	0/11 (0.0%)	**RR 1.67** (0.09 to 32.33) ^16^	not estimable	⨁◯◯◯ VERY LOW	CRITICAL
Risk of HCV (Allison, 2012) [11]												
1	observational study	serious ^8^	not serious	not serious	serious ^15^	none	19/469 (4.1%)	1/217 (0.5%)	**RR 8.79** (1.18 to 65.25) ^16^	36 more per 1000 (from 1 more to 296 more)	⨁◯◯◯ VERY LOW	CRITICAL
Risk of HCV (Kaldor, 1992) [15]												
1	observational study	serious ^9^	not serious	not serious	serious ^15^	none	7/220 (3.2%)	1/210 (0.5%)	**RR 6.9** (0.83 to 150)	28 more per 1000 (from 1 fewer to 710 more)	⨁◯◯◯ VERY LOW	CRITICAL
Risk of HCV (Murphy, 2000) [17]												
1	observational study	serious ^10^	not serious	not serious	serious ^15^	none	9/758 (1.2%)	2/1039 (0.2%)	**aOR 1** (0.3 to 3)	0 fewer per 1000 (from 1 fewer to 4 more)	⨁◯◯◯ VERY LOW	CRITICAL
Risk of HCV (Neal, 1994) [18]												
1	observational study	serious ^11^	not serious	not serious	serious ^15^	none	2/35 (5.7%)	0/150 (0.0%)	**aRR 7** (0.9 to 5.5)	not estimable	⨁◯◯◯ VERY LOW	CRITICAL
Risk of HCV (Serfaty, 2003) [21]												
1	observational study	serious ^12^	not serious	not serious	serious ^15^	none	3/35 (8.6%)	0/35 (0.0%)	**RR 7** (0.37 to 130.69) ^16^	not estimable	⨁◯◯◯ VERY LOW	CRITICAL
Risk of HCV (Tullen, 1993) [23]												
1	observational study	serious ^13^	not serious	not serious	serious ^15^	none	0/68 (0.0%)	0/103 (0.0%)	not estimable	not estimable	⨁◯◯◯ VERY LOW	CRITICAL
Risk of HCV (Van der Poel, 1991) [24]												
1	observational study	serious ^14^	not serious	not serious	serious ^15^	none	1/12 (8.3%)	2/113 (1.8%)	**OR 5.05** (0.42 to 60.2)	66 more per 1000 (from 10 fewer to 503 more)	⨁◯◯◯ VERY LOW	CRITICAL

aOR: adjusted odds ratio; aRR: adjusted risk ratio; HBV: hepatitis B virus; HCV: hepatitis C virus; HIV: human immunodeficiency virus; MSM: men who have sex with men; OR: odds ratio; RR: risk ratio; TTI: transfusion transmissible infections

1 Inappropriate eligibility criteria (random donors are a mix of male and female, while all homosexuals included are male); control for confounding unclear (not mentioned in article); other limitations: homosexuals were solicited via the press, other donors were random samples)

2 Random samples with young, minority and first-time donor oversampled; possible recall bias; the possible difference between respondents and non-respondents is not known

3 Control for confounding unclear (not mentioned in the article); no control group was included (e. g. states where no change in deferral policy was implemented): several blood safety-related interventions were implemented during the 10-year period covered by this study; there is a potential decline in the number of HIV-positive donors presenting to donate because of earlier HIV diagnosis in more recent times; before the implementation of the new policy, different states/territories had different policies (permanent deferral or 5 year deferral since last MSM contact) and the implementation of the new policy was not established at the same moment in each state/territory.

4 Control for confounding unclear (not mentioned in the article); no control group was included (e. g. states where no change in deferral policy was implemented)

5 First-time donations by infected persons (53.3%) were 1.7-fold higher than those among the control group (31.1%), which suggests that test-seeking donations may have been numerous.

6 Control for confounding unclear (not clear for which factors adjustment was made); questionnaire used to obtain information

7 Methods for exposure and outcome variables unclear (risk factors not determined in the same way for all cases); not controlled for confounding ((not clear if individuals with MSM as risk factor also have other risk factors)

8 Inappropriate eligibility criteria (all subjects had positive anti-HCV findings by enzyme immunoassay); not controlled for confounding

9 Inappropriate eligibility criteria (cases and controls not matched); not controlled for confounding (individuals with MSM as risk factor also have other risk factors); 5 out of 7 cases having homosexual contact also admitted personal drug use; questionnaire used

10 Control for confounding unclear (only adjusted for intravenous drug use, but unclear if this is sufficient)

11 Control for confounding unclear (only adjusted for intravenous drug use, but unclear if this is sufficient); questionnaire used to obtain information

12 Control for confounding unclear (only donors with no histories of transfusion or intravenous drug abuse, but unclear if this is sufficient); interview used to obtain information

13 Inappropriate eligibility criteria (cases and controls not matched; all subjects positive by ELISA); unclear if cases and controls received same questionnaire; control for confounding unclear (donors do not have former intravenous drug addiction, but unclear if this is sufficient); questionnaire used to obtain information

14 Inappropiate eligibility criteria (cases and controls not matched; all subjects positive by ELISA); not controlled for confounding (individuals with MSM as risk factor can also have other risk factors); interview used to obtain information

15 Low number of events

16 Calculations made by the reviewer(s) using Review Manager software

17 Not able to calculate confidence interval (data lacking)

18 Raw data calculated based on total number of participants and percentages

19 Raw data not available

20 Confidence interval of adjusted odds ratio not available, only p-value

#### Type 1 studies: comparison of TTI in MSM vs non-MSM donors

There is limited evidence (in number and quality) from two observational studies from 1985 and 2005, that male blood donors who had sex with other men after 1977 have a statistically significant higher risk of HIV-1, compared to male blood donors who did not have sex with other men [12,19]. The study of Alpaugh et al. found a risk ratio of 9.19, 95% CI 3.99–21.17 for the presence of antibody to hepatitis B core antigen (HBc) [12]. In this study the presence of antibodies to HBc was used to measure HBV infection. However this is an older test with poor specificity, which means that a high percentage of HBV negatives is incorrectly identified as HBV positives. Because results of this study are influenced by the test that was used to measure the outcome, we did not take the HBV data—in contrast to its HIV data—into account into our final conclusion [25]. For the presence of antibody to human T Cell Lymphotropic virus type III antigen (= HIV-1), the risk ratio was 6.90, 95% CI 2.10–22.68 for the initial reactive test, and 27.60, 95% CI 3.47–219.37 for the repeatable reactive test [12]. In the study by Sanchez et al., including 25,168 male donors, 6% of the donors who had sex with other men after 1977 had reactive screening test results, versus 1.7% of non-MSM donors, which was statistically significant. Analysis of the subpopulation between 1977 and 2010 indicated that the statistically significant difference was due to the MSM population with recent (< 12 months) sexual contact [19]. A statistically significant increased risk of infections could not be demonstrated for male blood donors who had sex with other men before 1977 (2.8% of MSM donors versus 1.7% of non-MSM donors) or for MSM more than one year ago since 1977, due to a large variability of the results (wide confidence intervals) [19].

#### Type 2 studies: comparison of TTI between different deferral strategies for MSM donors

In two studies, from 2010 and 2013, no statistically significant differences between types of deferral policy were measured [20,22]. In the first study, no statistically significant change in the number of HIV-positive male donors when applying a five versus a one year deferral period could be demonstrated. In the same study, the number of HIV-positive donors with MSM as a risk factor was also measured, however the number of male donors who disclosed risk factors is not known, and thus no conclusions can be made of these data [20]. In the second study, no statistically significant difference in the number of HIV-positive MSM donors could be demonstrated when a permanent deferral was changed to an individual risk assessment of sexual behaviours [22]. The results of these studies cannot be considered precise as the number of HIV-infected donors was too low [20,22].

#### Type 3 studies: comparison of infected vs non-infected blood donors for risk factors

Ten case-control studies investigated whether MSM is a risk factor for TTI such as HIV-1, HBV, or HCV in blood donors, comparing the risk profile of donors with TTI and matched healthy donors. All studies were performed when a permanent deferral strategy for MSM was in place, and thus any cases would have occurred despite this deferral strategy.

There is limited evidence from one observational study, published in 1994, about the correlation between MSM and the risk of HIV-1 infection in blood donors [13]. In this study it was shown that MSM is a statistically significant risk factor for HIV-1 infection in blood donors, reporting an odds ratio of 45, 95% CI 10.66–189.84. However, the results cannot be considered precise because of a low number of events (low number of donors with MSM as a risk factor among the cases and controls) [13].

For the risk of HBV infection, we found limited evidence from 2 observational studies from 1997 and 2001, but a statistically significant correlation could not be demonstrated between MSM and HBV infection in blood donors. The reason for this is a low number of events and a large variability of the results, reflected in wide confidence intervals [14,16].

The majority of the studies were searching for MSM as a risk factor for HCV in blood donors. A statistically significant increased risk of HCV-infection in blood donors could not be demonstrated for MSM in six of these studies (all performed in the early ‘90s and one in 2000), because of a low number of events (all studies) and/or a wide confidence interval [15,17,18,21,23,24]. In one more recent (2012) study it was shown that MSM blood donors have a statistically significant increased risk of HCV, reporting a risk ratio of 8.79, 95% CI 1.18–65.25, compared to non-MSM blood donors. However this risk ratio is a crude risk ratio, which represents a correlation between HCV data and MSM, not taking into account that the MSM donors could have other risk factors such as intravenous drug use or history of transfusions. Since the latter risk factors are confounding variables and no measures were taken to control for confounding in this study, this crude risk ratio, which we calculated ourselves based on the raw data provided in the study, was not used as part of the evidence base [11].

### Quality of the evidence

All studies included in this systematic review were observational studies, which results in an initial “low level of evidence” according to the GRADE approach, and there were no reasons to upgrade the level of evidence [10]. In order to determine the final level of evidence, first of all the risk of bias was assessed at the individual study level. In all studies, risk of bias was found because of limitations in study design or execution. For several studies there was no description of possible confounders nor of the method that was used to adequately control for confounding [11,12,14,15,20,22,24], or it was unclear if measures undertaken to adequately control for confounding were sufficient [17,18,21,23]. For 5 of the case-control studies inappropriate eligibility criteria were used for the selection of cases and controls [11,16,23,24]. In 5 studies questionnaires were used [14,15,18,19,23] and in 2 studies interviews were performed [21,24] to obtain information about risk factors, which is prone to recall bias. The studies by Seed et al. and Suligoi et al. are historically controlled, but groups where no change in deferral policy was implemented were not included [20,22]. In addition in the study by Seed et al., different states/territories had different policies (≥ 5 year deferral) before implementation of the new policy, and implementation of the new policy did not occur at the same moment in each state/territory [20]. In the study by Sanchez et al. and in the 11 case-control studies, donors who reported MSM since 1977 were donating despite being excluded from this practice. It cannot be excluded that these donors may have different characteristics from men with a history of MSM since 1977 who did not donate because of the current policy, but would if it were changed [11,13–19,21,23,24].

In addition to the risk of bias, the level of evidence was further downgraded because of imprecision (low number of events, large confidence intervals and lack of data). There was no reason to downgrade for inconsistency or indirectness. Overall, the strength of the body of evidence, as defined by the GRADE approach, is “very low” for all outcomes, which means that the estimates of effect are uncertain and further research is very likely to have an important impact on our confidence in the estimate of effect. An overview of how we obtained the level of evidence for all outcomes is shown in Table 2.

## Discussion

In this systematic review we searched for evidence on the association between MSM blood donors and TTI. We identified 14 studies: two studies directly comparing MSM with non-MSM donors, two studies comparing different types of deferral strategy for MSM and 10 studies comparing infected versus non-infected donors during a permanent deferral policy for MSM. Two studies however provided (statistically significant) data that were not taken into account to formulate our conclusions: the HBV data in the study of Alpaugh et al. were measured with an older diagnostic test with low specificity [12], and the HCV data in the study of Allison et al. were not taking into account any confounding variables [11].

We identified 3 studies looking at the risk of HIV-1 infection in MSM versus non-MSM donors. These studies showed a significant correlation between MSM and the risk of HIV-1 infection in blood donors. In addition, we found 3 studies looking at the risk of HBV infection and 7 studies looking at the risk of HCV infection, but none of these studies could demonstrate an increased risk of infection.

The available evidence is too limited to be able to unambiguously/clearly recommend a certain deferral policy, however one study suggests exclusion of MSM donors for at least 1 year after the last MSM contact. The latter however is based on very low level evidence from only 1 study and should therefore be interpreted with caution. This study showed that 8.3% of the donors who had sex with other men the last 12 months had reactive screening test results, versus 1.7% of non-MSM donors [19]. We found no evidence that MSM blood donors are at a higher risk of HBV and HCV infection. In the majority of the studies there was no information available about the period between the last MSM contact and the blood donation.

The main limitations of our analysis are:

1. the quality of the available evidence is defined according to the GRADE approach as “very low”, because of the study design (observational studies), a low number of infected individuals, a low number of donors with MSM as a risk factor, and/or large confidence intervals. Furthermore, the majority of the studies are older studies, including one study from 1985 and 7 studies from the early 1990s, which could result in bias since test specificity and sensitivity has improved significantly since then.
2. our analysis does not account for (non-)compliance in filling out the donor history questionnaire, as it is impossible to deduce from the studies what percentage of donors were honest about MSM behaviour, and of those who were not, what percentage were later forthright about this [26,27]. This in itself could compromise the quality of further studies. A recent study from Australia confirmed high compliance to a 12-month deferral for MSM, however compliance was calculated against the total population of male donors and not against the population of MSM donors, and no comparison between different deferral strategies was made [26]. High quality studies about the impact of different deferral strategies on non-compliance are currently lacking.
3. our systematic review did not capture unpublished surveillance data that are probably being collected by many blood services.
4. our analysis is limited to a certain geographic area, namely Western countries (Northern, Western, and Southern Europe, USA, Canada, Australia, New-Zealand) as defined in the selection criteria, since the populations in these countries are most relevant for our Blood Service. Because the epidemiology of STDs (which are also transfusion transmissible), sexual risk behaviour and STD prevention is different in developing countries, the results of this systematic review cannot be generalized.

Taking into account the limited evidence available, further higher quality research is necessary. There is a clear lack of studies directly comparing MSM and non-MSM donors, and studies comparing different deferral strategies. When comparing risk factors of infected and non-infected donors, it is important to describe the studied risk factors in detail and to mention the donor selection policy used.

The classic triad of evidence-based work consists of the best available evidence, complemented by expert opinion and by preference of the target population [28]. In the absence of strong evidence it is not surprising that expert opinion and preference (in this case both of patients and donors) play a greater role in determining policy than they would if the quality of the evidence were stronger. Donor preference (MSM group) is clear: many demand to be allowed to donate blood because they feel discriminated against [5]. Some do not contest exclusion on the basis of MSM, but rather the lifelong character of the exclusion, noting that it is applied to no other group on the basis of risk behaviour, except for, for example, intravenous drug users or commercial sex workers [29]. The preference of the patient population is also clear: they expect to receive the safest blood possible, but since most people are only future patients, they tend to be less informed and vocal, with the exception of haemophilia patient groups, who are both knowledgeable and very concerned about receiving safe blood.

Expert opinion mostly favours exclusion of MSM as the experts also take into account (1) evidence that is excluded in this systematic review because of its study type (e.g. uncontrolled studies); (2) evidence from outside the field of transfusion medicine such as higher prevalence of HIV and other TTI in MSM [1,2]; (3) that the right of the patient to the safest blood possible has predominance over the wish of a particular donor group to be allowed to donate blood [30]; (4) the tradition of the sector to make blood ever safer, following the precautionary principle (stating that, in the interest of public health, risk management action should be taken in the absence of certainty about risk) rather than the principles of health economics (not reimbursing measures that are not cost-effective) or “risk-based decision making” (implying that risk management actions should be proportionate to the level of demonstrated risk) [31,32].

## Conclusions

In summary, high-quality studies investigating the link between MSM blood donors and TTI are scarce. The available evidence suggests a link between MSM blood donors and HIV-1 infection. In one study it was shown that the significant correlation between MSM and HIV-1 infection was related to recent (< 12 months) MSM contact. This is however very low level evidence and more high quality studies are needed to be able to unambiguously/clearly recommend a certain deferral period for MSM. In absence of strong evidence, the length of the exclusion period mainly depends on regulators’ choices about whether the precautionary principle should continue to be applied in the blood banking sector or not, and on whether the estimated impact of exclusion/length of exclusion policy on non-compliance with the donor history questionnaire is greater than the gains made by applying the precautionary principle [26,27,33]. The scarcity of high-quality evidence within the field of transfusion medicine together with these choices may explain existing policy differences between countries [27,34].

## Supporting Information

S1 PRISMA Checklist PRISMA checklist. (PDF)

S1 File Search strategies. (PDF)

S1 Table List of studies excluded by at least one of the reviewers, including the reason for exclusion. (PDF)
