# Supplementary material for: Is Having Sex with Other Men a Risk Factor for Transfusion-Transmissible Infections in Male Blood Donors in Western Countries? A Systematic Review
Source: PLoS One. 2015 Apr 15;10(4):e0122523. doi: 10.1371/journal.pone.0122523 (PMC4398316; doi:10.1371/journal.pone.0122523)
Supplement: S1 File — (PDF) [file pone.0122523.s002.pdf]

Supporting information

Appendix 1 Search strategies

The following search formula was used for searching MEDLINE (PubMed interface):

1. "Homosexuality, Male"[Mesh] OR “men who have sex with men”[TIAB] OR “men having sex with men”[TIAB] OR “MSM”[TIAB] OR homosexual*[TIAB] OR bisexual*[TIAB] OR gay*[TIAB]

2. "Blood Donors"[Mesh] OR "Blood Transfusion"[Mesh] OR "Blood Safety"[Mesh] OR “blood”[TIAB] OR "transfusion"[TIAB] OR "Donor Selection"[Mesh] OR "Blood Banks"[Mesh] OR “deferral”[TIAB]

3. 1 AND 2

4. "Blood Donors"[Mesh] OR blood don*[TIAB] OR "Blood Banks"[Mesh] OR blood bank[TIAB] OR blood banks[TIAB] OR blood service*[TIAB] OR blood center*[TIAB] OR blood centre*[TIAB] OR transfusion service*[TIAB] OR transfusion cent*[TIAB]

5. "Sexually Transmitted Diseases"[Mesh] OR “STD”[TIAB] OR “STI”[TIAB] OR sexually transm*[TIAB] OR “transfusion transmissible”[TIAB] OR “TTVI”[TIAB] OR “TTVIs”[TIAB] OR “TTIs”[TIAB] OR “TTI”[TIAB] OR "Communicable Diseases, Emerging"[Mesh] OR “HIV”[Mesh] OR “human immunodeficiency virus”[TIAB] OR “HIV”[TIAB] OR “AIDS”[TIAB] OR “LAV”[TIAB] OR “Lymphadenopathy Associated Virus”[TIAB] OR “ARV”[TIAB] OR "HTLV-I Infections"[Mesh] OR "HTLV-II Infections"[Mesh] OR "Human T-lymphotropic virus 1"[Mesh] OR "Human T-lymphotropic virus 2"[Mesh] OR “HTLV”[TIAB] OR “lymphotropic virus”[TIAB] OR "Chlamydia trachomatis"[Mesh] OR “Chlamydia”[TIAB] OR "Hepatitis, Viral, Human"[Mesh] OR "Hepatitis Viruses"[Mesh] OR “hepatitis”[TIAB] OR “HCV”[TIAB] OR “HBV”[TIAB] OR "Treponema pallidum"[Mesh] OR “syphilis”[TIAB] OR “Treponema pallidum”[TIAB]

6. "Homosexuality, Male"[Mesh] OR MSM[TIAB] OR homosexual*[TIAB] OR bisexual*[TIAB] OR gay*[TIAB] OR “Sexual behavior”[Mesh] OR “Sexual partners”[Mesh] OR "Sex Factors"[Mesh] OR "Substance Abuse, Intravenous"[Mesh] OR “Tattooing”[Mesh] OR pierc*[TIAB] OR needle[TIAB] OR needles[TIAB] OR syringe[TIAB] OR syringes[TIAB] OR inject*[TIAB] OR drug[TIAB] OR drugs[TIAB] OR tattoo*[TIAB] OR sexual[TIAB] OR sex[TIAB] OR blood transfusion*[TIAB] OR partner[TIAB] OR partners[TIAB] OR "Risk Factors"[Mesh] OR "Risk Assessment"[Mesh] OR "Risk"[Mesh:NoExp] OR "Risk-Taking"[Mesh] OR “risk factor”[TIAB] OR “risk factors”[TIAB] OR deferrable risk*[TIAB] OR “UDR”[TIAB]

7. 4-6 AND

8. 3 OR 7

The following search formula was used in Embase (Embase.com interface):

1. 'male homosexual'/exp OR ‘men who have sex with men’:ab:ti OR ‘men having sex with men’:ab:ti OR ‘MSM’:ab:ti OR homosexual*:ab:ti OR bisexual*:ab:ti OR gay*:ab:ti

2. 'blood donor'/exp OR 'blood transfusion'/exp OR 'blood safety'/exp OR blood:ab:ti OR transfusion:ab:ti OR 'donor selection'/exp OR 'blood bank'/exp OR deferral:ab:ti

3. 1 AND 2

4. 'blood donor'/exp OR (blood NEXT/1 don*):ab:ti OR 'blood bank'/exp OR ‘blood bank’:ab:ti OR ‘blood banks’:ab:ti OR (blood NEXT/1 service*):ab:ti OR (blood NEXT/1 (center* OR centre*)):ab:ti OR (transfusion NEXT/1 service*):ab:ti OR (transfusion NEXT/1 cent*):ab:ti

5. 'sexually transmitted disease'/exp OR 'STD':ab:ti OR 'STI':ab:ti OR (sexually NEXT/1 transm*):ab:ti OR 'transfusion transmissible':ab:ti OR 'TTVI':ab:ti OR 'TTVIs':ab:ti OR 'TTI':ab:ti OR 'TTIs':ab:ti OR 'emerging':ab:ti OR 'Human immunodeficiency virus'/exp OR 'Human immunodeficiency virus infection'/exp OR 'human immunodeficiency virus':ab:ti OR 'HIV':ab:ti OR 'AIDS':ab:ti OR 'LAV':ab:ti OR 'Lymphadenopathy Associated Virus':ab:ti OR 'ARV':ab:ti OR 'Human T cell leukemia virus infection'/exp OR 'Human T cell leukemia virus 1'/exp OR 'Human T cell leukemia virus 2'/exp OR 'HTLV':ab:ti OR 'lymphotropic virus':ab:ti OR 'Chlamydia trachomatis'/exp OR 'Chlamydia':ab:ti OR 'chlamydiasis'/exp OR 'hepatitis virus'/exp OR 'hepatitis':ab:ti OR 'HCV':ab:ti OR 'HBV':ab:ti OR 'Treponema pallidum'/exp OR 'syphilis':ab:ti OR 'Treponema pallidum':ab:ti

6. 'homosexuality'/exp OR 'sexual behavior'/exp OR 'sexuality'/de OR ‘MSM’:ab:ti OR homosexual*:ab:ti OR bisexual*:ab:ti OR gay*:ab:ti OR 'substance abuse'/exp OR 'tattooing'/exp OR pierc*:ab:ti OR ‘needle’:ab:ti OR ‘needles’:ab:ti OR ‘syringe’:ab:ti OR ‘syringes’:ab:ti OR inject*:ab:ti OR ‘drug’:ab:ti OR ‘drugs’:ab:ti OR tattoo*:ab:ti OR ‘sexual’:ab:ti OR ‘sex’:ab:ti OR (blood NEXT/1 transfusion*):ab:ti OR ‘partner’:ab:ti OR ‘partners’:ab:ti OR 'risk'/de OR 'risk factor'/exp OR 'risk assessment'/exp OR 'high risk behavior'/exp OR 'infection risk'/exp OR 'risk reduction'/exp OR ‘risk factor’:ab:ti OR ‘risk factors’:ab:ti OR (deferrable NEXT/1 risk*):ab:ti OR ‘UDR’:ab:ti

7. 4-6 AND

8. 3 OR 7

The following search formula was used in Cochrane Central Register of Controlled Trials:

1. MeSH descriptor Homosexuality, Male explode all trees OR (“men who have sex with men”):ti,ab,kw OR (“men having sex with men”):ti,ab,kw OR (MSM):ti,ab,kw OR (homosexual*):ti,ab,kw OR (bisexual*):ti,ab,kw OR (gay*):ti,ab,kw

2. MeSH descriptor Blood Donors explode all trees OR MeSH descriptor Blood Transfusion explode all trees OR MeSH descriptor Blood Safety explode all trees OR (blood):ti,ab,kw OR (transfusion):ti,ab,kw OR MeSH descriptor Donor Selection explode all trees OR MeSH descriptor Blood Banks explode all trees OR (deferral):ti,ab,kw

3. 1 AND 2

4. [mh “Blood Donors”] OR (blood NEXT don*):ti,ab,kw OR [mh “Blood Banks"] OR “blood bank” :ti,ab,kw OR “blood banks”:ti,ab,kw OR (blood NEXT service*):ti,ab,kw OR (blood NEXT center*):ti,ab,kw OR (blood NEXT centre*):ti,ab,kw OR (transfusion NEXT service*):ti,ab,kw OR (transfusion NEXT cent*):ti,ab,kw

5. [mh "Sexually Transmitted Diseases"] OR “STD”:ti,ab,kw OR “STI”:ti,ab,kw OR (sexually NEXT transm*):ti,ab,kw OR “transfusion transmissible”:ti,ab,kw OR “TTVI”:ti,ab,kw OR “TTVIs”:ti,ab,kw OR “TTIs”:ti,ab,kw OR “TTI”:ti,ab,kw OR [mh "Communicable Diseases, Emerging"] OR [mh HIV] OR “human immunodeficiency virus”:ti,ab,kw OR “HIV”:ti,ab,kw OR “AIDS”:ti,ab,kw OR “LAV”:ti,ab,kw OR “Lymphadenopathy Associated Virus”:ti,ab,kw OR “ARV”:ti,ab,kw OR [mh "HTLV-I Infections"] OR [mh "HTLV-II Infections"] OR [mh "Human T-lymphotropic virus 1"] OR [mh "Human T-lymphotropic virus 2"] OR “HTLV”:ti,ab,kw OR “lymphotropic virus”:ti,ab,kw OR [mh "Chlamydia trachomatis"] OR “Chlamydia”:ti,ab,kw OR [mh "Hepatitis, Viral, Human"] OR [mh "Hepatitis Viruses"] OR “hepatitis”:ti,ab,kw OR “HCV”:ti,ab,kw OR “HBV”:ti,ab,kw OR [mh "Treponema pallidum"] OR “syphilis”:ti,ab,kw OR “Treponema pallidum”:ti,ab,kw

6. 4 AND 5

7. 3 OR 6

The following search strategy was used in Cinahl:

1. MH "Homosexuals, Male" OR TI “men who have sex with men” OR AB “men who have sex with men” OR TI “men having sex with men” OR AB “men having sex with men” OR TI “MSM” OR AB “MSM” OR TI homosexual* OR AB homosexual* OR TI bisexual* OR AB bisexual* OR TI gay* OR AB gay*

2. MH "Blood Donors" OR MH "Blood Transfusion" OR MH "Blood Banks" OR TI "Donor Selection" OR AB “Donor Selection” OR TI "Blood Safety" OR AB “Blood Safety” OR TI “blood” OR AB “blood” OR TI “transfusion” OR AB “transfusion” OR TI “deferral” OR AB “deferral”

3. 1 AND 2

4. MH “Blood Donors” OR TI “blood don*” OR AB “blood don*”OR MH “Blood Banks" OR TI “blood bank” OR TI “blood banks” OR AB “blood bank” OR AB “blood banks” OR TI “blood service*” OR AB “blood service*” OR TI “blood center*” OR TI “blood centre*” OR AB “blood center*” OR AB “blood centre*” OR TI “transfusion service*” OR AB “transfusion service*” OR TI “transfusion cent*” OR AB “transfusion cent*”

5. MH "Sexually Transmitted Diseases+" OR TI “STD” OR TI “STI” OR TI “sexually transm*” OR TI “transfusion transmissible” OR TI “TTVI” OR TI “TTVIs” OR TI “TTIs” OR TI “TTI” OR MH "Communicable Diseases, Emerging" OR MH “Human immunodeficiency virus” OR TI “human immunodeficiency virus” OR TI “HIV” OR TI “AIDS” OR TI “LAV” OR TI “Lymphadenopathy Associated Virus” OR TI “ARV” OR MH "HTLV-I Infections" OR MH "Human T-lymphotropic virus 2" OR TI “HTLV” OR TI “lymphotropic virus” OR MH "Chlamydia trachomatis" OR TI “Chlamydia” OR MH "Hepatitis, Viral, Human+" OR MH "Hepatitis Viruses" OR TI “hepatitis” OR TI “HCV” OR TI “HBV” OR TI “syphilis” OR TI “Treponema pallidum” OR AB “STD” OR AB “STI” OR AB “sexually transm*” OR AB “transfusion transmissible” OR AB “TTVI” OR AB “TTVIs” OR AB “TTIs” OR AB “TTI” OR AB “human immunodeficiency virus” OR AB “HIV” OR AB “AIDS” OR AB “LAV” OR AB “Lymphadenopathy Associated Virus” OR AB “ARV” OR AB “HTLV” OR AB “lymphotropic virus” OR AB “Chlamydia” OR AB “hepatitis” OR AB “HCV” OR AB “HBV” OR AB “syphilis” OR AB “Treponema pallidum”

6. MH "Homosexuals, Male" OR TI “MSM” OR TI “homosexual*” OR TI “bisexual*” OR TI “gay*” OR MH “Sexuality+” OR MH “Sexual partners” OR MH "Sex Factors" OR MH "Substance Abuse, Intravenous" OR MH “Tattooing” OR TI “pierc*” OR TI “needle” OR TI “needles” OR TI “syringe” OR TI “syringes” OR TI “inject*” OR TI “drug” OR TI “drugs” OR TI “tattoo*” OR TI “sexual” OR TI “sex” OR TI “blood transfusion*” OR TI “partner” OR TI “partners” OR MH "Risk Factors" OR MH "Risk Assessment" OR MH "Risk-Taking behaviour+" OR TI “risk factor” OR TI “risk factors” OR TI “deferrable risks” OR TI “UDR” OR AB “men who have sex with men” OR AB “men having sex with men” OR AB “MSM” OR AB “homosexual*” OR AB “bisexual*” OR AB “gay*” OR AB “pierc*” OR AB “needle” OR AB “needles” OR AB “syringe” OR AB “syringes” OR AB “inject*” OR AB “drug” OR AB “drugs” OR AB “tattoo*” OR AB “sexual” OR AB

“sex” OR AB “blood transfusion*” OR AB “partner” OR AB “partners” OR AB “risk factor” OR AB “risk factors” OR AB “deferrable risks” OR AB “UDR”

7. 4-6 AND

8. 3 OR 7

The following search strategy was used in Web of Science:

1. TI=(“men who have sex with men”) OR TS=(“men who have sex with men”) OR TI=(“men having sex with men”) OR TS=(“men having sex with men”) OR TI=(MSM) OR TS=(MSM) OR TI=(homosexual*) OR TS=(homosexual*) OR TI=(bisexual*) OR TS=(bisexual*) OR TI=(gay*) OR TS=(gay*)

2. TI=(“blood donors”) OR TS=(“blood donors”) OR TI=(“blood transfusion”) OR TS=(“blood transfusion”) OR TI=(“donor selection”) OR TS=(“donor selection”) OR TI=(“blood banks”) OR TS=(“blood banks”) OR TI=(“blood safety”) OR TS=(“blood safety”) OR TI=(blood) OR TS=(blood) OR TI=(transfusion) OR TS=(transfusion) OR TI=(deferral) OR TS=(deferral)

3. 1 AND 2

4. TS=(“blood don*”) OR TS=(“blood bank”) OR TS=(“blood banks”) OR TS=(“blood service*”) OR TS=(“blood center*”) OR TS=(“blood centre*”) OR TS=(“transfusion service*”) OR TS=(“transfusion cent*”)

5. TS=(“STD”) OR TS=(“ STI”) OR TS=(“sexually transm*”) OR TS=(“transfusion transmissible”) OR TS=(“TTVI”) OR TS=(“TTVIs”) OR TS=(“TTIs”) OR TS=(“TTI”) OR TS=(“human immunodeficiency virus”) OR TS=(“HIV”) OR TS=(“AIDS”) OR TS=(“LAV”) OR TS=(“Lymphadenopathy Associated Virus”) OR TS=(“ARV”) OR TS=(“HTLV”) OR TS=(“lymphotropic virus”) OR TS=(“Chlamydia”) OR TS=(“hepatitis”) OR TS=(“HCV”) OR TS=(“HBV”) OR TS=(“syphilis”) OR TS=(“Treponema pallidum”)

6. TS=(“MSM”) OR TS=(“homosexual*”) OR TS=(“bisexual*”) OR TS=(“gay*”) OR TS=(“pierc*”) OR TS=(“needle”) OR TS=(“needles”) OR TS=(“syringe”) OR TS=(“syringes”) OR TS=(“inject*”) OR TS=(“drug”) OR TS=(“drugs”) OR TS=(“tattoo*”) OR TS=(“sexual”) OR TS=(“sex”) OR TS=(“blood transfusion*”) OR TS=(“partner”) OR TS=(“partners”) OR TS=(“risk factor”) OR TS=(“risk factors”) OR TS=(“risk-taking”) OR TS=(“risk assessment”) OR TS=(“deferrable risks”) OR TS=(“UDR”)

7. 4-6 AND

8. 3 OR 7
