# Supplementary material for: Is Having Sex with Other Men a Risk Factor for Transfusion-Transmissible Infections in Male Blood Donors in Western Countries? A Systematic Review
Source: PLoS One. 2015 Apr 15;10(4):e0122523. doi: 10.1371/journal.pone.0122523 (PMC4398316; doi:10.1371/journal.pone.0122523)
Supplement: S1 Table — (PDF) [file pone.0122523.s003.pdf]

Supporting information

Appendix 2 List of studies excluded by at least one of the reviewers, including the reason for exclusion

First author	Publication date	Title	Type of exclusion	Reason for exclusion
Aach	1980	Post-transfusion hepatitis: analysis of risk factors	Intervention	Did not include MSM as a risk factor
Adams	1984	AIDS and blood donors	Design	Editorial
Agomoh	2011	Giving blood... 'Why Can't Men Who Have Sex With Men Donate Blood?' Viewpoint, October 2010	Design	Opinion piece
Ahmetagic	2006	Hepatitis C infection in risk groups	Intervention	Did not include MSM as a risk factor
Allain	2013	Infectivity of blood products from donors with occult hepatitis B virus infection	Intervention	No risk factors studied
Allen	1985	Risk-Factors for Htlv-Iii Infection in Volunteer Blood-Donors with Anti-Htlv-Iii	Design	Risk factors not specified
Alonso	2009	Preventing transfusion-transmitted HIV infection in Latin America and the Caribbean: issues associated with blood donor interviews and sex between men	Population	Latin-America
Alter	1997	Hepatitis C in asymptomatic blood donors	Intervention	Risk factors not assessed in control group
Altes	1995	Blood donors seropositive for type 1 human immunodeficiency virus (HIV-1). Epidemiologic characteristics and clinical course	Design	No control group
Ambrozaitis	1995	Hepatitis C in Lithuania: incidence, prevalence, risk factors and viral genotypes	Design	No control group
Anderson	1992	HIV antibody testing and posttest counseling in the United States: Data from the 1989 National Health Interview Survey	Design	Survey
Anderson	2009	Quantitative estimate of the risks and benefits of possible alternative blood donor deferral strategies for men who have had sex with men	Design	Modelling study
Andre	1977	HBs-antigen and blood transfusion (author's transl)	Intervention	No risk factors studied
Araujo	1998	The blood bank and hepatitis G	Intervention	No risk factors studied
Arcieri	1998	Retrospective studies of blood transfusion-associated AIDS infection: The Italian 'look-back' program 1990-1995	Intervention	Risk factors not assessed at the level of blood donors
Ashour	2009	Decreasing the risk of transfusion transmitted infections (TTIS) in the Egyptian Blood Transfusion Services	Intervention	MSM not studied
Atrah	1996	Unexplained hepatitis C virus antibody seroconversion in established blood donors	Design	No control group
Austin	2000	Prevalence of hepatitis C virus seropositivity among hospitalized US veterans	Intervention	Did not include MSM as a risk factor
Aymard	1989	Epidemiologic study of HIV serology in blood donors from 5 departments in northeastern France (1985-1989)	Intervention	Risk factors not assessed in control group
Aymard	1990	Serum antibodies against hepatitis C: study of 4,100 blood donors from the northeast of France	Intervention	No risk factors studied
Azar	1993	Post-transfusional anti-HCV-negative, non-A, non-B hepatitis. (I) a prospective clinical and epidemiological survey	Intervention	Risk factors not assessed at the level of blood donors
Balogun	2003	Risk factors, clinical features and genotype distribution of diagnosed hepatitis C virus infections: a pilot for a sentinel laboratory-based surveillance	Design	No control group
Barbara	1992	Hepatitis C and blood transfusion	Design	Narrative review
Barbara	2000	Prevalence of antibodies to HTLV in blood donors in north London	Design	Letter
Barker	1980	Posttransfusion hepatitis: epidemiology, experimental studies and US perspective	Design	Narrative Review
Barker	1982	Viral hepatitis: lessons from blood donors	Design	Narrative Review

Barna	1996	Hepatitis C virus antibody in the serum of blood donors	Design	No control group
Bastard	2006	Giving blood: an individual right or the expression of a social responsibility? The donors' position in the debates on blood transfusion	Design	Narrative review
Bateman	2000	Should gay men donate blood?	Design	Opinion piece
Bateman	2006	Gay 'militant' stays bloody minded	Design	Narrative text
Bates	2000	Identification of risk factors in blood donors found to have HIV infection	Intervention	Risk factors not assessed in control group
Bauer	2010	The right to give blood	Design	Letter
Bauer	2010	The right to give blood [2]	Design	Letter
Baumgarten	1990	AIDS and blood transfusion	Intervention	Risk factors not assessed in control group
Bayer	1983	Gays and the stigma of bad blood	Design	Opinion piece
Beal	1968	A prospective study of south Australian blood donors	Intervention	No risk factors studied
Beauplet	2003	Medical selection of blood donors: risk prevention for the recipient	Design	Flowcharts for donor selection
Behr-Gross	2013	Contributions of the Council of Europe's Blood Transfusion Steering Committee to the determination of rules for the selection of donors of blood and blood components and the study of sexual behaviors having an impact on blood safety	Design	Opinion piece
Behrmann	2011	Do Canadian researchers have 'blood on their hands'?	Design	Narrative text
Behrmann	2013	A Gift That Some Cannot Give: The Ethical Significance of the Ban on Gay/Bisexual Men as Blood Donors	Design	Opinion piece
Belanger	2013	If the permanent deferral were lifted would men who have sex with men want to donate blood, and if so, who would be eligible?	Intervention	Questionnaire to determine interest of MSM to donate
Benjamin	2011	Deferral of males who had sex with other males	Design	Survey about deferral policies worldwide
Berner	2011	The making of a risk object: AIDS, gay citizenship and the meaning of blood donation in Sweden in the early 1980s	Design	Narrative review
Bizzaro	1992	Serum Alanine Aminotransferase Levels Among Volunteer Blood-Donors - Effect of Sex, Alcohol Intake and Obesity	Outcome	Only ALT as outcome
Björkman	2001	A case-control study of transmission routes for GB virus C/ hepatitis G virus in Swedish blood donors lacking markers for hepatitis C virus infection	Outcome	Hepatitis G virus
Bönig	2012	Sufficient blood, safe blood: can we have both?	Design	Opinion piece
Bouchardeau	2000 Jul	GB virus type C/HGV markers in HCV RNA-positive French blood donors: correlation with HCV genotypes and risk factors	Intervention	Risk factors not assessed in control group
Boulton	2006	Analysis of complaints received by the UK blood services concerning the policy barring homosexual men from donating blood	Outcome	No infections measured as outcome
Brant	2007	Increase in recently acquired syphilis infections in English, Welsh and Northern Irish blood donors	Design	No control group
Brant	2011	Hepatitis B and residual risk of infection in English and Welsh blood donors, 1996 through 2008	Design	No control group
Brooks	2004	The rights of blood recipients should supersede any asserted rights of blood donors	Design	Opinion piece
Brooks	2009	Should men who have ever had sex with men be allowed to give blood?	Design	Comment
Brooks	2011	Blood donation in men who have sex with men	Design	Comment
Bruckova	2007	HIV/AIDS in the Czech Republic, 2006	Design	Report
Bujandric	2006	Hepatitis B in blood donors: Analysis of testing results in the period 2000-2005 in blood transfusion institute novi sad	Intervention	Risk factors not assessed in control group
Burger	2000	Hepatitis B virus	Design	Narrative Review
Burrell	1985	The blood story. Part II. AIDS--current concepts and implications for blood transfusion services and nursing staff	Design	Guidelines
Busch	1991	Evaluation of Screened Blood Donations for Human-Immunodeficiency-Virus Type-1 Infection by Culture and Dna Amplification of Pooled Cells	Intervention	MSM not studied

Busch	1994	Screening of blood donors for idiopathic CD4+ T-lymphocytopenia	Intervention	MSM not studied
Busch	1994	HIV and blood transfusions: focus on seroconversion	Intervention	No risk factors studied
Busch	1995	Time course of detection of viral and serologic markers preceding human immunodeficiency virus type 1 seroconversion: implications for screening of blood and tissue donors	Design	Diagnostic study
Busch	1996	Factors influencing human immunodeficiency virus type 1 transmission by blood transfusion	Intervention	Factors influencing transmission from donor to recipient
Busch	1997	Value and cost-effectiveness of screening blood donors for antibody to hepatitis B core antigen as a way of detecting window-phase human immunodeficiency virus type 1 infections. The HIV Blood Donor Study Group	Design	No control group
Busch	2003	Current and emerging infectious risks of blood transfusions	Design	Narrative Review
Busch	2006	Lessons and opportunities from epidemiologic and molecular investigations of infected blood donors	Design	Editorial
Cable	1997	HIV infected blood donors have a long interdonation interval - Implications for HIV risk assessment	Intervention	No risk factors studied
Cable	2006	Risk factors reported by HIV infected blood donors, 2001-2005	Intervention	Risk factors not assessed in control group
Cammarota	1991	Aids - Evaluation of 26,424 Blood-Donors and 1,282 High-Risk Subjects by Immunoassay	Population	Argentina
Caplan	2010	Blood stains--why an absurd policy banning gay men as blood donors has not been changed	Design	Editorial
Carbone	2013	Analysis of HIV HCV and HBV sierovirological positiveness among the blood donors of the Lazio region and the south part of Tuscany	Intervention	No risk factors studied
Cardoso	1998	Identification of infection routes for HCV in blood donors from Germany	Intervention	Did not include MSM as a risk factor
Carlson	1996	The risk of HIV transmission by screened blood	Design	Comment on Lackritz 1995
Carneiro-Proietti	1999	HIV-(1/2) indeterminate western blot results: follow-up of asymptomatic blood donors in belo horizonte, minas gerais, brazil	Population	Brazil
Carney	2013	Association of tattooing and hepatitis C virus infection: A multicenter case-control study	Population	General population; no blood donors
Carvalho	1987	HIV antibodies in beggar blood donors in Rio de Janeiro, Brazil	Population	Brazil
Cascio	2011	Defining sexual contact in donor educational materials: Considerations for the debate on men who have sex with men	Intervention	Educational material to define sexual contact
Casey	2011	Illicit Regulation: A Framework for Challenging the Procedural Validity of the 'Gay Blood Ban'	Design	Narrative text
Caspari	1987	HIV-specific antibody among voluntary blood donors in Lower Saxony (FRG)	Intervention	Risk factors not assessed in control group
Caspari	1997	Non-specific and specific anti-HCV results correlated to age, sex, transaminase, rhesus blood group and follow-up in blood donors	Intervention	Comparison between different diagnostic tests
Celum	1994	Risk factors for repeatedly reactive HIV-1 EIA and indeterminate western blots. A population-based case-control study	Population	Mixed population, including blood donors, and people form high-risk testing sites (e.g. Sexually Transmitted Diseases Clinic)
Chamberland	1994	Human immunodeficiency virus infection among health care workers who donate blood	Intervention	Risk factors not assessed in control group
Chan	2007	Disentangling the stigma of HIV/AIDS from the stigmas of drugs use, commercial sex and commercial blood donation -	Outcome	Levels of stigma were measured

		a factorial survey of medical students in China		
Chanchlani	2013	New research leads to end of blood ban	Design	News item
Chapman	1989	AIDS. Blood transfusions: reducing the risk	Design	Narrative Review
Chataing	1989	Abstention and exclusion of donors exposed to the risk of HIV infection. Report of the meeting of 9 March 1989	Design	Report
Chatlynne	1998	Detection and titration of human herpesvirus-8-specific antibodies in sera from blood donors, acquired immunodeficiency syndrome patients, and Kaposi's sarcoma patients using a whole virus enzyme-linked immunosorbent assay	Intervention	MSM not studied
Chattopadhya	1998	Evaluation of epidemiological and serological predictors of human immunodeficiency virus type-1 (HIV-1) infection among high risk professional blood donors with western blot indeterminate results	Intervention	Did not include MSM as a risk factor
Chave	1988	HIV-positive blood donors: An epidemiologic survey	Intervention	Risk factors not assessed in control group
Chiavetta	1992	Reported Hiv Risk-Factors in Volunteer Blood-Donors	Design	Short report about older study
Chiavetta	1992	Prevalence of antibody to human T-cell lymphotropic virus type I/II in people of Caribbean origin in Toronto	Population	Carribean households in Toronto
Chicot	1974	Clinical, epidemiological and biochemical study of a population of blood donors carrying Australia antigen and hepatitis B	Design	No control group
Chou	2004	Screening for Hepatitis C Virus Infection: A Review of the Evidence for the U.S. Preventive Services Task Force	Intervention	No risk factors studied
Cleary	1988	Sociodemographic and behavioral characteristics of HIV antibody-positive blood donors	Intervention	Risk factors not assessed in control group
Cleary	1991	Trends in sociodemographic and behavioral characteristics of HIV antibody-positive blood donors	Intervention	Risk factors not assessed in control group
Cleary	1993	Depressive Symptoms in Blood-Donors Notified of Hiv-Infection	Design	No control group
Cochran	2009	Self-reported altruistic and reciprocal behaviors among homosexually and heterosexually experienced adults: implications for HIV/AIDS service organizations	Design	Survey
Conry-Cantilena	1992	Risk-Factors for Hepatitis-C Virus-Infection in A United-States Urban Blood-Donor Population	Intervention	Did not include MSM as a risk factor
Conry-Cantilena	1996	Routes of infection, viremia, and liver disease in blood donors found to have hepatitis C virus infection	Intervention	Did not include MSM as a risk factor
Contreras	1985	Blood donors at high risk of transmitting the acquired immune deficiency syndrome	Design	Questionnaire; no data available
Contreras	1985	Acquired immune deficiency syndrome and blood transfusion	Design	Narrative text
Courouce	1987	Results of the first 9 months of testing for anti-HIV antibodies in blood donors in France]	Population	Insufficient info on control group
Courouce	1990	Prevalence and epidemiologic features of subjects found to be infected with HIV when donating blood. CTS of the 'Retrovirus' Study Group of the Societe Nationale de Transfusion Sanguine]	Population	Insufficient info on control group
Courouce	1993	HIV seropositivity in blood donors from 1990 to 1992: prevalence, estimation of residual risk of transfusion-related infections and epidemiology]	Population	Insufficient info on control group
Courouce	1996	Transfusion-transmitted viral infections [3]	Design	Letter
Courouce	2000	Transmitted-transfusion infections. Screening in French blood donations from 1996 to 1998	Intervention	Risk factors not assessed in control group
Courtois	1999	Risk behavior among blood donors: efficacy of a new questionnaire	Intervention	MSM not studied
Coutinho	1998	The Amsterdam Cohort Studies on HIV infection and AIDS	Design	Description of cohort
Cowan	1996	Behavioural risk factors for HIV infection amongst blood donors in London	Population	Blood donors versus general population
Cowell	1985	AIDS and community heath issues	Design	Narrative Review

Crawford	1994	Prevalence and epidemiological characteristics of hepatitis C in Scottish blood donors	Intervention	Risk factors not assessed in control group
Curran	1984	The acquired immunodeficiency syndrome associated with transfusions: the evolving perspective	Design	No control group
Curran	1984	Acquired immunodeficiency syndrome (AIDS) associated with transfusions	Design	Narrative text
Curran	1985	Epidemiologic aspects of acquired immunodeficiency syndrome (AIDS) in the United States: cases associated with transfusions	Design	No control group
da Silva	1998	Identification of infection routes for HCV in blood donors from German	Intervention	Did not include MSM as a risk factor
Dagnra	2002	Prevalence and risk of HCV transmission after screening for HIV and HBV in blood donnors [1]	Design	Letter
Davidson	2009	Human immunodeficiency virus 1 subtypes detected in Scottish blood donors	Design	Insufficient info on control group
Davison	2011	A re-evaluation of the risk of transfusion-transmitted HIV prevented by the exclusion of men who have sex with men from blood donation in England and Wales, 2005-2007	Design	Modelling study
Davison	2013	The risk of transfusion-transmitted HIV from blood donations of men who have sex with men, 12 months after last sex with a man: 2005-2007 estimates from England and Wales	Design	Modelling study
de Almeida	2007	Risk factors for human immunodeficiency virus infection among blood donors in Sao Paulo, Brazil, and their relevance to current donor deferral criteria	Population	Brazil
de Almeida	2009	Profile of blood donors with serologic tests reactive for the presence of syphilis in Sao Paulo, Brazil	Population	Brazil
De Alwis	2010	Prevalence of human immunodeficiency virus among Sri Lanken blood donors	Population	Sri Lanka
De Andres-Medina	1993	HIV seropositivity of blood donations in Spain: A 4-year surveillance (1988-1991) [1]	Intervention	Risk factors not assessed in control group
de Guevara	2002	Hepatitis C prevalence and risk factors among a blood donors cohort	Population	Blood donors in Mexico
de la Fuente	2009	Human immunodeficiency virus testing uptake and risk behaviours in Spain	Population	Households in Spain
de Saussure	1993	Human immunodeficiency virus type 1 nucleic acids detected before p24 antigenemia in a blood donor	Design	Case study
de Statio	1990	Screening for anti-human T-lymphotropic virus antibody in blood donors and polytransfused patients in Apulia (Italy)	Intervention	No risk factors studied
Delage	1999	Risk factors for acquisition of hepatitis C virus infection in blood donors: results of a case-control study	Population	Risk factor is "Sexual activity with same sex partner", not clear if only men are included
Delarocque-Astagneau	2007	An incident case-control study of modes of hepatitis C virus transmission in France	Intervention	Did not include MSM as a risk factor
Derrick	1984	AIDS and the use of blood components and derivatives: The Canadian perspective	Design	Narrative review
Desmyter	1986	AIDS and blood transfusion	Design	Letter
Dodd	1985	Donor screening and epidemiology	Design	Report
Dodd	1989	Transfusion and AIDS	Design	Narrative text
Doll	1991	Human immunodeficiency virus type 1-infected blood donors: Behavioral characteristics and reasons for donation	Design	No control group
Doll	1992	Homosexually and nonhomosexually identified men who have sex with men: A behavioral comparison	Outcome	No infections as outcome
Domok	2001	Factors and facts in Hungarian HIV/AIDS epidemic, 1985-2000	Design	Report
Donegan	1990	Infection with human immunodeficiency virus type 1 (HIV-1) among recipients of antibody-positive blood donations	Population	Recipients of antibody positive blood donations
Dougan	2004	HTLV infection in England and Wales in 2002--results from an enhanced national surveillance system	Design	No control group
Dudok de Wit	1994	HIV-positive blood donors, 1985-1993	Design	No control group

Dupont	1993	Prevention of risks related to blood transfusions	Design	Narrative text
Eble	1992	Resolution of infection status of human immunodeficiency virus (HIV)-seroindeterminate donors and high-risk seronegative individuals with polymerase chain reaction and virus culture: absence of persistent silent HIV type 1 infection in a high-prevalence area	Intervention	PCR and virus culture to detect HIV+ blood donors
Eble	1993	Determination of human T lymphotropic virus type by polymerase chain reaction and correlation with risk factors in northern California blood donors	Intervention	Risk factors not assessed in control group
Eder	2009	Selection Criteria to Protect the Blood Donor in North America and Europe: Past (Dogma), Present (Evidence), and Future (Hemovigilance)	Design	Narrative review
Elghouzzi	1995	Transmission of hepatitis B virus by HBV-negative blood transfusion	Design	Case study
Elghouzzi	2000	Hepatitis C virus: routes of infection and genotypes in a cohort of anti-HCV-positive French blood donors	Intervention	Risk factors not assessed in control group
Elira-dokekias	1988	AIDS and blood transfusion (preventive strategy in the African region)	Design	Narrative review
Elliott	2003	European Court of Human Rights accepts blood donor screening based on sexual orientation	Design	Opinion piece
Enbom	2000	Antibodies to human herpesvirus 8 latent and lytic antigens in blood donors and potential high-risk groups in Sweden: Variable frequencies found in a multicenter serological study	Intervention	No search for risk factors
Esteban	1991	High rate of infectivity and liver disease in blood donors with antibodies to hepatitis C virus	Intervention	Did not include MSM as a risk factor
Estermann	1992	Epidemiology of HIV infections in Switzerland and the German Federal Republic--contribution of official surveillance systems, of anonymous testing and of blood donor screening	Population	General population and blood donors
Fearon	2009	A follow-up study on Canadian blood donors testing positive for hepatitis B anti-core antibody (anti-HBc)	Intervention	Risk factors not assessed in control group
Fejza	2009	Prevalence of HBV and HCV among blood donors in Kosovo	Design	No control group
Feorino	1984	Lymphadenopathy associated virus infection of a blood donor--recipient pair with acquired immunodeficiency syndrome	Design	Case study
Ferroni	1992	Hiv-1 Infection in Italian Blood-Donors During A 5 Year Surveillance	Intervention	Risk factors not assessed in control group
Fiebig	1998	Safety of the blood supply	Design	Narrative review
Fiedler	1994	Anti-HIV rates in blood donations in Europe and the safety of the national blood supplies [8]	Design	Letter
Fielding	2006	Risk-behavior reporting by blood donors with an automated telephone system	Population	Potential blood donors
Fisher	2010	Sex and blood: a deeper exploration of discrimination in the FDA blood donor policy	Design	Comment on Galarneau
Flanagan	2012	How should we assess risk behaviour when determining donor deferral? Reflections on the MSM deferral	Design	Narrative review
Forsythe	2010	Blood, tissue and organ safety: Upcoming initiatives from the governments' advisory committee	Design	Narrative text
Fournel	1991	Blood transfusions: improved safety	Design	Interview
Fox	2010	The expressive dimension of donor deferral	Design	Comment
Franklin	2007	Is there a right to donate blood? Patient rights; donor responsibilities	Design	Narrative review
Frosner	1975	Prevalence of hepatitis B antibody in blood donors from south-western Germany (author's transl)	Design	Narrative text
Galarneau	2010	Blood donation, deferral, and discrimination: FDA donor deferral policy for men who have sex with men	Design	Narrative review
Galarneau	2010	A response to commentaries on "Blood donation, deferral, and discrimination"	Design	Opinion piece
Gallagher	2013	Advancing risk assessment for emerging infectious diseases	Design	Report

		for blood and blood products: proceedings of a public workshop		
Garmaise	2002	Canadian news. Blood donor screening practices criticized	Design	Opinion piece
Garmaise	2006	Lifetime ban on blood donations from gay men to continue	Design	Opinion piece
Garmaise	2006	Gay man launches suit over refusal to accept blood donation	Design	Opinion piece
Gathof	1989	Results of anti-HIV testing of blood donors 1985-1988	Design	Narrative text
Germain	2002	Men who have had sex with men and blood donation: is it time to change our deferral criteria?	Design	Narrative review
Germain	2003	The risks and benefits of accepting men who have had sex with men as blood donors	Design	Modelling study
Germain	2013	Allowing blood donation from men who had sex with men more than 5 years ago: A model to evaluate the impact on transfusion safety in Canada	Design	Modelling study
Giulivi	1992	Anti-hepatitis C virus (HCV) screening at a Canadian Red Cross center: significance of a positive c100 HCV enzyme-linked immunosorbent assay	Intervention	Risk factors not assessed in control group
Gluck	1990	HIV prevalence in blood donors in urban and in rural areas of the Federal Republic of Germany	Population	Urban versus rural blood donors; no control group
Gluck	1990	HIV study of the German Red Cross of West Germany 1985-1989: prevalence/incidence and mode of infection, retrospective studies	Intervention	Risk factors not assessed in control group
Go	2011	The attitude of Canadian university students toward a behavior-based blood donor health assessment questionnaire	Intervention	Testing two alternative questionnaires
Goedert	1985	Blood donation by persons at high risk of AIDS	Design	Letter
Goldman	2011	Donor understanding and attitudes about current and potential deferral criteria for high-risk sexual behavior	Intervention	MSM not studied; outcome: attitude
Goodnough	1999	Transfusion medicine. First of two parts--blood transfusion	Design	Narrative review
Goodnough	2003	Risks of blood transfusion	Design	Narrative review
Goodrick	1992	History of Previous Drug Misuse in Hcv-Positive Blood-Donors	Design	Narrative text
Goodrick	1994	Hepatitis C (HCV)-positive blood donors in south-west England: a case control study	Intervention	Risk factor studied: homosexual/bisexual contact
Goodwin	1999	Retrospective donor profiling: a tool for enhancing blood safety	Intervention	No risk factors studied
Goritsas	2000	HCV infection in the general population of a Greek island: prevalence and risk factors	Intervention	Did not include MSM as a risk factor
Gracie	1985	AIDS--again	Design	Narrative text
Gray	1997	Anger over HIV questions cancels blood clinic at Ottawa university	Design	Comment
Grenfell	2011	Views and experiences of men who have sex with men on the ban on blood donation: a cross sectional survey with qualitative interviews	Outcome	No infections as outcome
Guillard	1990	The medical interview motivated by the discovery of markers of viral hepatitis permits the identification, in blood donors, of behavior at risk for HIV infection]	Population	Insufficient info on control group
Gunson	1988	HIV antibody screening of blood donations in the United Kingdom	Population	Insufficient info on control group
Gunson	1991	Screening of blood donations for HIV-1 antibody: 1985-1991	Intervention	Risk factors not assessed in control group
Gurtler	1994	Safety from HIV infection of preserved blood from Germany	Design	Editorial
Habibi	1986	AIDS and blood transfusion	Design	Narrative review
Hamers	2003	HIV in central and eastern Europe	Design	Epidemiologic report
Heck	2013	Comparison of seroprevalence to human immunodeficiency virus (HIV), hepatitis c (HCV) and hepatitis b (HBV) in tissue donors and first-time blood donors in North Texas	Intervention	No risk factors studied
Heintges	1997	Hepatitis C virus: epidemiology and transmission	Design	Narrative review
Hellstrom	1985	AIDS: recent events	Design	Narrative text
Herget	2005	Egale granted intervenor status as party in Canadian blood donor case	Design	News

Hewitt	2013	Human T-lymphotropic virus lookback in NHS Blood and Transplant (England) reveals the efficacy of leukoreduction	Design	Epidemiologic report
Hitzler	1996	Prevalence of HTLV-I/II in repeat blood donors in Germany	Intervention	No risk factors studied
Hladik	2003	Most highly exposed seronegative men lack HIV-1-specific, IFN-gamma-secreting T cells	Population	Uganda
Hollan	1990	Results of longitudinal immunological surveillance of individuals directly or indirectly infected by a single HIV seropositive donor	Design	Case series
Hollinger	2009	Hepatitis B virus traceback and lookback: Factors to consider	Design	Narrative review
Holloman	1993	Evaluation of testing algorithms following the use of combination HIV-1/HIV-2 EIA for screening purposes	Intervention	HIV screening method
Holzberger	1992	Second generation anti-HCV test: seroprevalence in hemodialysis patients and blood donors	Intervention	HCV screening method
Huang	2012	Risk factors for hepatitis B and C virus among chinese blood donors	Population	Blood donors with Hepatitis B and C infection
Hurley	1998	Bad blood	Design	Opinion piece
Hurley	2009	Bad blood: gay men and blood donation	Design	Opinion piece
Hurley	2011	UK lifts lifetime ban on gay men giving blood	Design	Opinion piece
Iraporda	1995	HCV-positive blood donors. Risk factors	Design	Letter
Irving	1993	Hepatitis C in blood donors	Design	Letter
Isbister	1985	Strategies for avoiding or minimizing homologous blood transfusion: a sequel to the AIDS scare	Design	Narrative review
Isbister	1988	The paradigm shift in blood transfusion	Design	Narrative text
Jamil	2013	Prevalence of Antibodies to the Hepatitis C Virus Among Arab and Chaldean Americans in Southeast Michigan, Usa	Population	General population
Jett	1983	Acquired immunodeficiency syndrome associated with blood-product transfusions	Design	Case study
Jimenez Del Bianco	2010	Epidemiological data of syphilis positive donors	Design	Survey
Josefson	2000	FDA declines to lift ban on homosexual men as blood donors	Design	Opinion piece
Jovicic	2010	Analysis of the Frequency of Positive Results to Transfusion Transmissible Diseases Markers in Blood Donors at the Blood Transfusion Institute of Serbia from 2006 to 2008	Intervention	Risk factors not assessed in control group
Joyce	1989	Recruiting blood donors in view of AIDS: what ambulatory care facilities need to understand	Design	Recruitment of donors
Jullien	1990	Lessening the risk of transmission of hepatitis C virus by labile blood products: a role for indirect markers?	Intervention	HCV screening method
Kagi	1989	Prevalence of cytomegalovirus, hepatitis B and HIV-1 antibodies in healthy blood donors, hospital patients, kidney transplant recipients, i.v. drug addicts and homosexuals	Population	Healthy blood donors vs homosexuals
Kaldor	1991	Human immunodeficiency virus antibodies in sera of Australian blood donors: 1985-1990	Intervention	Risk factors not assessed in control group
Kalish	1986	Voluntary deferral of blood donations and HTLV-III antibody positivity	Design	Letter
Kalish	1987	Voluntary self-exclusion to reduce transmission of AIDS by blood transfusion	Design	Letter
Kaplan	1985	AIDS: blood donor studies and screening programs	Design	Narrative review
Katz	1992	Non-A non-B hepatitis and hepatitis C virus	Design	Narrative text
Kay	1989	Retention of regular blood donors	Design	Letter
Kerzman	2006	Risk factors for HCV seropositivity - case-control study on blood donors: immigrants from the former Soviet Union as compared to other blood donors	Population	Societ Union immigrants in Israel
Khabbaz	1992	Indeterminate HTLV serologic results in U.S. blood donors: are they due to HTLV-I or HTLV-II?	Intervention	Did not include MSM as a risk factor
Kiser	2013	Gay blood drive attempts to change FDA policy	Design	Opinion piece
Kitchen	1998	Hepatitis B and blood safety	Design	Narrative text
Klein	1996	Blood donors with antibody to hepatitis C virus [5]	Design	Letter
Kleinman	1986	Anti-HTLV III ELISA and Western blot testing in a blood donor population: implications for donor notification	Design	Blood donors with HTLV

Kleinman	1994	Interview studies of HIV seropositive donors	Design	Reasons why HIV positives give blood
Kleinman	2012	The National Heart, Lung, and Blood Institute retrovirus epidemiology donor studies (Retrovirus Epidemiology Donor Study and Retrovirus Epidemiology Donor Study-II): twenty years of research to advance blood product safety and availability	Design	Narrative review.
Kleinman	2013	The National Heart, Lung, and Blood Institute Recipient Epidemiology and Donor Evaluation Study (REDS-III): A research program striving to improve blood donor and transfusion recipient outcomes	Design	Narrative review
Klugman	2010	Blood donation and its metaphors	Design	Comment
Koch	1993	Blood donors as the sentinel population for sexually transmissible diseases--results of a sociodemographic study and survey of sexual behavior	Outcome	HIV status not measured
Koch	2013	Evolution of residual risk for HIV, HCV and HBV from 1991 to 2010, in blood donations of the Centro Hospitalar S. Joao, EPE, Porto, Portugal	Intervention	No risk factors studied
Kolho	1992	Risk factors for hepatitis C virus antibody positivity in blood donors in a low-risk country	Intervention	Did not include MSM as a risk factor
Kolins	1986	The continued risk of transfusion-transmitted AIDS	Design	Narrative text
Kondro	2001	Panel rejects u-turn on gay blood donor ban	Design	Opinion piece
Koppelman	2004	Diversity and origin of hepatitis B virus in Dutch blood donors	Design	No control group
Koulentaki	1999	Prevalence of hepatitis B and C markers in volunteer blood donors in Crete. A 5-year study	Intervention	No risk factors studied
Krekulova	2001	Genotypic distribution of TT virus (TTV) in a Czech population: evidence for sexual transmission of the virus	Population	Does not compare infected vs uninfected blood donors
Kucirka	2011	Risk of window period HIV infection in high infectious risk donors: systematic review and meta-analysis	Design	No control group
Kucirka	2011	Risk of window period hepatitis-C infection in high infectious risk donors: systematic review and meta-analysis	Design	No control group
Kuhnl	1987	Investigations of donors and recipients of HIV antibody-positive blood units	Design	No control group
Kuhnl	1987	HTLV III/LAV antibody screening: Interlaboratory trial of the German blood donor services	Intervention	Evaluation of antibody screening test
Kuhnl	1988	HIV and blood transfusion in West Germany	Design	Narrative text
Kulathunge	2009	Whole blood donor deferral pattern in a Sri Lankan population	Population	General population; Sri Lanka
Kuriyan	1984	Effects of AIDS on various aspects of blood banking	Design	Narrative text
Lam	2011	Prevalence and demographic determinants of hepatitis B surface antigen (HBSAG) and anti-HBCORE in first-time U.S. blood donors, 2004-2009	Other	Not available; oral presentation (available as a study: Murphy)
Lang	1989	Seroepidemiologic studies of cytomegalovirus and Epstein-Barr virus infections in relation to human immunodeficiency virus type 1 infection in selected recipient populations. Transfusion Safety Study Group	Population	Insufficient info on control group
Laperche	2003	Recent HCV infections in blood donors (1994-2001)	Design	Report
Larkin	2000	South Africa reels from blood-donation debacle	Design	Opinion piece
Lee	1991	Relative prevalence and risk factors of HTLV-I and HTLV-II infection in US blood donors	Intervention	Risk factors not assessed in control group
Lefrere	1990	Interviews with HIV seropositive subjects identified at the time of blood donation: consequences for pre-donation interviews	Intervention	Risk factors not assessed in control group
Lefrere	1991	No evidence of frequent human immunodeficiency virus type 1 infection in seronegative at-risk individuals	Population	Insufficient info on control group
Lefrere	1992	Interviews with anti-HIV-positive individuals detected through the systematic screening of blood donations: Consequences on predonation medical interview	Intervention	Risk factors not assessed in control group
Lefrere	1996	Interviews of individuals diagnosed as anti-human	Intervention	Risk factors not

		immunodeficiency virus-positive through the screening of blood donations in the Paris area to 1994: reflections on the selection of blood donors		assessed in control group
Leiss	2008	Men having sex with men donor deferral risk assessment: an analysis using risk management principles	Design	Opinion piece
Leon	1998	How to reduce the prevalence of HIV-positive blood donors	Design	No control group
Leon	1998	Serum hepatitis B markers in blood donors in Venezuela. What do they mean?	Design	No control group
Leon de Gonzalez	1994	HIV and blood donors: past, present and future	Design	No control group
Levine	1985	Screening blood: public health and medical uncertainty	Design	Narrative text
Li	2010	HIV among plasma donors and other high-risk groups in Henan, China	Population	Survey in high risk groups; China
Lieshout-Krikke	2014	Surface antigen-negative hepatitis B virus infection in Dutch blood donors	Intervention	No risk factors studied
Linden	1988	An estimate of blood donor eligibility in the general population	Population	General population
Lizsewski	2014	The rates, perceptions, and willingness of men who have sex with men to donate blood	Intervention	No risk factors studied
London	1984	Epidemiologic lessons from viral hepatitis B	Design	Narrative text
Lopez	2004	Hepatitis C seroprevalence in accepted versus deferred blood-donor candidates evaluated by medical history and self-exclusion form	Population	Mexican blood donors
Lucky	2013	Trends in transfusion-transmissible infections among Australian blood donors from 2005 to 2010	Intervention	Risk factors not assessed in control group
MacLennan	1994	A study of anti-hepatitis C positive blood donors: the first year of screening	Intervention	Did not include MSM as a risk factor
Magni	1987	Hepatitis D virus (HDV) infection in Lombardia based on the observations of the transfusion services. Hepatitis B Study Group of the Italian Association of Immunohaematology and Blood Transfusion	Design	Narrative text
Maisonneuve	1991	Circulating antibodies to hepatitis C virus: Analysis of risk factors in seropositive blood donors from metropolitan France	Intervention	MSM not studies as separate risk factor
Mansuy	2011	Hepatitis E virus antibodies in blood donors, France	Intervention	Did not include MSM as a risk factor
Margaritis	2011	Nucleic acid amplification testing of australian blood donors highlights the prevalence of occult hepatitis B virus infection in a HBV low prevalence population	Intervention	Risk factors not assessed in control group
Martin	1992	Hepatitis C: Evaluating the seropositive blood donor	Design	Commentary
Martucci	2010	Negotiating exclusion: MSM, identity, and blood policy in the age of AIDS	Design	Narrative review
Mast	1997	Prevalence of and risk factors for antibody to hepatitis E virus seroreactivity among blood donors in Northern California	Intervention	Did not include MSM as a risk factor
McClelland	2005	Appropriateness and safety of blood transfusion	Design	Narrative text
McCullough	2000	Nucleic acid amplification testing of blood donors for transfusion-transmitted infectious diseases	Intervention	Not about MSM
McLindon	1995	Hepatitis C-related chronic liver disease among asymptomatic blood donors in the north west of England	Design	No control group
Means	2011	Blood risks from gay donors... AN=2011307775	Design	Letter
Menitove	1985	Transfusion associated AIDS	Design	Narrative text
Millar	2010	Assessing infectious risk in blood donors to inform evidence based health screening policy	Design	Survey
Miller	1983	Acquired immunodeficiency syndrome (AIDS) and blood products	Design	Narrative text
Miller	1985	Potential liability for transfusion-associated AIDS	Design	Narrative text
Minga	2005	Behavior assessment of blood donors facing the risk of HIV infection, Abidjan, Cote D'Ivoire, 2001-2002	Population	Ivory Coast
Minola	1998	Epidemiology, viremia, and liver disease in blood donors found to have hepatitis C virus infection: A prospective study	Intervention	Did not include MSM as a risk factor
Miyakawa	2009	Epidemiological study of hepatitis B virus genotype a in	Population	Japan

		Japanese blood donors		
Molijn	1997	Risk factors and anti-HBc reactivity among first time blood donors	Design	No control group
Montalto	1994	Antibodies to hepatitis C virus and histological pattern in Sicilian blood donors	Design	No control group
Moore	1972	Incidence of hepatitis B antigen and antibody among Canadian volunteer blood donors	Design	Letter
Moore	1995	Blood donors who are repeatedly reactive for hepatitis C virus on enzyme immunoassay and positive on recombinant immunoblot assay: evidence of failure to identify some risk factors	Intervention	Did not include MSM as a risk factor
Moore	1998	Anti-HBc in blood donors	Design	Letter
Moore	2002	Antibodies to hepatitis B core in Danish blood donors: a surrogate marker for 'high-risk' behaviour?	Design	Letter
Muller	2001	The detection of hepatitis C virus in South Hungary	Intervention	Did not include MSM as a risk factor
Murphy	1995	A Case-Control Study of Risk-Factors for Infection with Human T-Lymphotropic Viruses Type-I and Type-Ii (Htlv-I and Htlv-Ii) Among Us Blood-Donors	Intervention	Did not include MSM as a risk factor
Murphy	1996	Biological and clinicopathological features associated with hepatitis C virus type 5 infections	Intervention	Risk factors not assessed in control group
Murphy	1996	Demographic determinants of hepatitis C virus seroprevalence among blood donors	Intervention	MSM not studied
Murphy	1997	Criteria for screening blood donors: science or politics?	Design	Comment
Murphy	1998	Risk factors for hepatitis C virus (HCV) infection in US blood donors - Lack of association with drug inhalation	Intervention	Did not include MSM as a risk factor
Murphy	1999	Evidence among blood donors for a 30-year-old epidemic of human T lymphotropic virus type II infection in the United States	Intervention	No MSM in population
Murphy	2004	Higher human T lymphotropic virus (HTLV) provirus load is associated with HTLV-I versus HTLV-II, with HTLV-II subtype A versus B, and with male sex and a history of blood transfusion	Intervention	No MSM in population
Murphy	2009	Liver disease, antiviral treatment and behavioral risk factors in a retrospective cohort of HCV seropositives detected at the time of blood donation	Intervention	Did not include MSM as a risk factor
Murphy	2009	Hepatitis C virus prevalence and clearance Among U.S. Blood donors, 2006-2007: Associations With birth cohort, multiple pregnancies and Body mass index	Intervention	Did not include MSM as a risk factor
Murphy	2010	Hepatitis C virus prevalence and clearance among US blood donors, 2006-2007: associations with birth cohort, multiple pregnancies, and body mass index	Intervention	Did not include MSM as a risk factor
Murphy	2011	HTLV-1 and -2 seroprevalence among United States blood donors, 2000-2009	Intervention	Risk factors not assessed in control group
Murphy	2012	Prevalence and demographic determinants of hepatitis B virus in first-time U.S. blood donors, 2004-2009	Intervention	Did not include MSM as a risk factor
Musto	2008	Estimating the risk of blood donation associated with HIV risk behaviours	Design	Modelling study
Mutimer	1993	Hepatitis C virus antibody positive blood donors	Intervention	Risk factors not assessed in control group
Nandi	1994	Detection of HIV-1, HBV and HCV antibodies in blood donors from Surat, western India	Population	India
Nategh	1975	Incidence of hepatitis B antigen in acute viral hepatitis, professional blood donors and drug addicts	Population	Iran
Nau	2006	Of homosexuality and blood donation (1)	Design	Letter
Nau	2006	Of homosexuality and blood donation (2)	Design	Letter
Nau	2011	Homosexuality and blood donation 3	Design	Opinion piece
Nau	2011	Homosexuality and blood donation 4	Design	Opinion piece
Neal	1994	Injecting Drug-Use, Health-Care Work and Tattoos, Risk-Factors for Hepatitis-C Infection in United-Kingdom Blood-Donors	Other	Poster abstract containing preliminary data,

				which were later published (Neal 1994, included study) (this was confirmed after contacting the author)
Neill	2010	Don't ask, don't tell . . . and don't donate	Design	Editorial
Niederhauser	2008	Occult hepatitis B infection in blood donors	Design	Survey
Nishioka	1991	Blood transfusion and HIV infection. Current status in the world and Japan	Population	Japan
No authors listed (PMID:6583516)	1984	AIDS and blood transfusions	Design	Statements
No authors listed (PMID:4046110)	1985	Leads from the MMWR. Revised definition of persons who should refrain from donating blood and plasma	Design	Statements
No authors listed (PMID:3944969)	1986	Leads from the MMWR. Update: acquired immunodeficiency syndrome--Europe	Design	Report
No authors listed (PMID:3123906)	1987	Human immunodeficiency virus infection in the United States: a review of current knowledge	Design	Narrative Review
No authors listed (PMID:3119981)	1987	Human immunodeficiency virus infection in the United States	Design	Narrative Review
No authors listed (PMID:3336168)	1988	Leads from the MMWR. Human immunodeficiency virus infection in the United States	Design	Narrative Review
No authors listed (PMID:1911168)	1991	Hepatitis C (parenterally transmitted non-A, non-B hepatitis)	Design	Report
No authors listed (PMID:7533587)	1995	Hepatitis C and blood transfusion	Design	Report
No authors listed (PMID:17868593)	2007	Monitoring HIV prevalence in blood donations in Europe	Design	Editorial
No authors listed (PMID: 18613339)	2008	Policy. University halts blood drives, cites donation discrimination	Design	News
No authors listed (Cinahl Acc Nr: 2000065234)	2000	Constitutional right of gay men to donate blood	Population	South-Africa
No authors listed (Cinahl Acc Nr: 2000041478)	2000	Equal rights panel can't fine plasma bank for rejecting gay donor	Design	Opinion piece
No authors listed (Cinahl Acc Nr: 2000071861)	2000	Letters... picture for the article 'Constitutional rights of GAY men to donate blood' (May 2000 issue)	Design	News item
No authors listed (Cinahl Acc Nr: 19955080)	2006	Students campaign against bar on bisexual and gay blood donors	Design	News item

No authors listed (PMID: 17319018)	2007	Blood donations. New rules may shrink ranks of blood donors: AIDS Policy Law 2007	Design	Opinion piece
No authors listed (PMID: 20225506)	2009	Constitutionality of gay blood donor ban challenged in court: HIV AIDS Policy Law Rev 2009	Design	Opinion piece
No authors listed (Cinahl Acc Nr: 2010469083)	2009	Call for end to ban on gay men giving blood	Design	Opinion piece
No authors listed (WOS:000280 172600008)	2010	Should gay men be allowed to donate blood? A new study has called for a review of the lifetime ban on gay blood donors in Canada	Design	Narrative review
No authors listed (WOS:000280 039200003)	2010	Should men who have sex with men be allowed to donate blood? A new study has called for a review of the lifetime ban in Canada	Design	Narrative review
No authors listed (Cinahl Acc Nr: 66917864)	2011	Clinical digest. Gay men say new blood donation rules need to be explained clearly	Design	Opinion piece, news item
No authors listed (Cinahl Acc Nr: 2011296287)	2011	Lifting of blood ban could improve HIV awareness	Design	Opinion piece
No authors listed (Cinahl Acc Nr: 60413396)	2011	Nurses want blood donation ban lifted	Design	Opinion piece
Norda	2011	Risk behaviours having an impact on blood donor Management	Design	Survey
Nordbo	2000	Prevalence of GB virus C (also called hepatitis G virus) markers in Norwegian blood donors	Intervention	Did not include MSM as a risk factor
Novak	1972	Australia antigen survey in Hungary. I. Healthy blood donors	Intervention	Did not include MSM as a risk factor
Nubling	2001	Human T-cell lymphocytotrophic virus prevalence in German blood donors and 'at-risk' groups	Design	Letter
Nusbacher	1987	Evaluation of a confidential method of excluding blood donors exposed to human immunodeficiency virus: studies on hepatitis and cytomegalovirus markers	Intervention	No risk factors studied
O'Brien	2008	Declining hepatitis C rates in first-time blood donors: insight from surveillance and case-control risk factor studies	Intervention	Did not include MSM as a risk factor
O'Brien	2009	Human T lymphotropic virus in Canadian blood donors	Intervention	Only abstract available (poster), but nothing mentioned about MSM, and MSM not studied in other studies from O'Brien
O'Brien	2013	The epidemiology of human T-cell lymphotropic virus types I and II in Canadian blood donors	Intervention	Did not include MSM as a risk factor
Offergeld	2011	Is the deferral of men who have sex with men (MSM) from donating still appropriate?	Design	Opinion piece
Offergeld	2012	HIV, HCV, HBV and syphilis surveillance among blood donors in Germany 2008-2010	Design	Epidemiologic report
Ogilvie	2011	Lift ban on gay blood donors?	Design	Opinion piece
Ono-Nita	2006	Hepatitis C - Facts in numbers	Design	Editorial
Operskalski	1989	Epidemiologic background of blood donors with antibody to human T-cell lymphotropic virus. Transfusion Safety Study Group	Design	Insufficient info on control group
Orton	2000	Identification of recent risk factors among blood donors confirmed to be HCVNAT reactive, anti-HCV non-reactive	Intervention	Did not include MSM as a risk factor
Orton	2004	Risk factors for HCV infection among blood donors confirmed to be positive for the presence of HCV RNA and	Intervention	Did not include MSM as a risk factor

		not reactive for the presence of anti-HCV		
Parker	2010	The right to give blood [1]	Design	Comment
Parks	1991	Human T-cell lymphotropic virus infection among blood donors in south Florida. The Transfusion Safety Study Group	Intervention	Did not include MSM as a risk factor; no control group
Payne	2011	Gay men in stable relationships still face a ban on donations... (news September 14)	Design	Opinion piece
Pazin	1989	How safe is the blood supply?	Design	Narrative text
Pearson	2011	NHS blood and transplant welcomes donor review... ban on gay and bisexual men donating blood (features March 23)	Design	Opinion piece
Peate	2011	HIV: still a serious issue for gay men as the blood donation ban is eased	Design	Comment
Peate	2011	Review of gay donor policy should be robust and fair... From the heart (features March 23)	Design	Opinion piece
Perkins	1985	Transfusion-associated AIDS	Design	Narrative text
Peterman	1987	Transfused-associated acquired immunodeficiency syndrome	Design	Narrative text
Petersen	1991	Human immunodeficiency virus type 1-infected blood donors: Epidemiologic, laboratory, and donation characteristics	Design	Donors with HIV
Petersen	1993	Hiv Transmission Through Blood, Tissues, and Organs	Design	Narrative review
Phillips	2008	Does blood discriminate?	Design	Comment
Pillonel	2010	Deferral from donating blood of men who have sex with men: Impact on the risk of HIV transmission by transfusion	Design	Modelling study
Pillonel	2011	Access to blood donation of men who have sex with men and impact on the risk of HIV transmission by transfusion: international overview	Design	Narrative review
Pillonel	2012	Deferral from donating blood of men who have sex with men: impact on the risk of HIV transmission by transfusion in France	Design	Modelling study
Politis	1999	The prevalence of anti-HTLV-I-II in blood donors in Greece. A multi- center study	Intervention	Risk factors not assessed in control group
Politis	2009	Look back programme for HIV seropositive blood donors in Hellas	Intervention	Risk factors not assessed in control group
Politis	2013	Surveillance of HIV infection in the general population and in blood donors in Greece: The impact of economic crisis on risk behaviours	Intervention	Risk factors not assessed in control group
Polizzotto	2008	Reducing the risk of transfusion-transmissible viral infection through blood donor selection: the Australian experience 2000 through 2006	Intervention	Risk factors not assessed in control group
Prati	1997	The incidence and risk factors of community-acquired hepatitis C in a cohort of Italian blood donors	Intervention	Did not include MSM as a risk factor
Pruett	2010	Feces in our food, viruses in our organs: Donor surveillance, organ transplantation and the risk for disease transmission	Design	Editorial
Quaglio	2008	Prevalence and risk factors for viral hepatitis in the Kosovarian population: implications for health policy	Intervention	Did not include MSM as a risk factor
Quigley	2010	Don't let stigma govern health	Design	Opinion piece
Quinton	1988	Plagues and morality	Design	Narrative text
Quiros	1998	GBV-C RNA presence in several high-risk groups of Spain	Intervention	No risk factors studied
Rall	1995	Epidemiology of hepatitis C virus infection	Design	Narrative text
Rassam	1991	Epidemiology and transmission of hepatitis C infection	Design	Narrative text
Reesink	1992	Risk-Factors for Hcv Infection in Blood-Donors	Intervention	No risk factors studied
Regine	2013	Low perception of sexual behaviours at risk for human immunodeficiency virus infection among blood donors who call the AIDS/STI Help Line in Italy	Outcome	No infection measured
Reik	2006	Unique donor-suitability issues	Design	Narrative review
Reinhardt	1969	Frequency of hepatitis-suspect indications in blood donors from various collection areas	Intervention	No risk factors studied
Richer	1975	Hepatitis B antigen in Montreal blood donors: childhood institutionalization as an epidemiologic factor	Design	No control group

Roberts	2010	Is donor selection appropriate and non-discriminatory? i.e. Should men who have sex with men be allowed to donate blood	Design	Opinion piece
Roehr	2009	Should men who have ever had sex with men be allowed to give blood?	Design	Opinion piece
Roerh	2009	Should men who have ever had sex with men be allowed to give blood? Yes	Design	Opinion piece
Roman	2010	Viewpoint. Why can't men who have sex with men donate blood? It's time to end the FDA's ban	Design	Opinion piece
Romano	2013	Hepatitis B virus infection among first-time blood donors in Italy: prevalence and correlates between serological patterns and occult infection	Intervention	No risk factors assessed
Roome	1975	Incidence of Herpesvirus hominis antibodies among blood donor populations	Population	Hospital patients, blood donors, prison populations
Rosenberg	2013	Acute hepatitis B in blood donors over a 5-year period in England and North Wales: who is getting infected?	Intervention	Risk factors not assessed in control group
Ross	1985	Characteristics of homosexual men who donate blood	Design	No control group
Saleun	1991	Anti-Hbc Antibodies in Blood-Donors - Distribution According to Sex and Age	Intervention	No risk factors studied
Sampietro	1996	Hepatitis GB virus C [1]	Design	Editorial
Saracco	1987	HDV infection from blood and blood products	Intervention	No risk factors studied
Saura	1997	Screening for markers of blood-borne diseases in donated units collected in France from 1993 to 1995	Intervention	Risk factors not assessed in control group
Scaccetti	2013	Prevalence of viral infections in the donor population at the terni immunohematology and transfusion service (SIT) in the three-year period 2010-2012	Intervention	No risk factors studied
Schell	2004	Extent of physician's explanation regarding transfusions--risk of infection in donor blood transfusions must be considered	Design	Narrative text
Schneider	1977	Hepatitis-B surface antigen and antibody in Black and White patients with venereal diseases	Intervention	Did not include MSM as a risk factor
Schreiber	1997	Risk factors for human T-cell lymphotropic virus types I and II (HTLV-I and -II) in blood donors: the Retrovirus Epidemiology Donor Study. NHLBI Retrovirus Epidemiology Donor Study	Intervention	Did not include MSM as a risk factor
Schreiber	2006	Marginal recruitment impact of potential alterations to FDA deferral criteria for men who have sex with men - Reply	Design	Reply on Sanchez et al.
Schricker	1987	AIDS from the viewpoint of transfusion medicine	Design	Narrative text
Schroter	1999	Prevalence of TTV viremia among healthy subjects and individuals at risk for parenterally transmitted diseases in Germany	Intervention	No risk factors studied
Schutt	1990	Epidemiological aspects of HIV infection in blood donors	Design	No control group
Scott	1985	Effect of the acquired immune deficiency syndrome (AIDS) on blood transfusion and donation patterns in the State of Minnesota	Design	Survey
Seage	1988	Patterns of blood donations among individuals at risk for AIDS, 1984	Design	No control group
Seed	2013	Low prevalence of non-compliance to the current 12 month deferral for male-to-male sex in australia	Intervention	Questionnaire to determine compliance
Seed	2014	Compliance with the current 12-month deferral for male-to-male sex in Australia	Intervention	Questionnaire to determine compliance
Seidl	1990	Hepatitis C virus (HCV) antibodies in German blood donors--a pilot study	Intervention	No risk factors studied
Sheikh	2013	Seroprevalence of Hepatitis B and C Infections among Healthy Volunteer Blood Donors in the Central California Valley	Design	Epidemiologic report
Sheikh	2013	Seroprevalence of Hepatitis B and C Infections among Healthy Volunteer Blood Donors in the Central California Valley	Intervention	No risk factors studied
Sheldon	1981	Panel: 'present use of blood and blood products.'	Design	Panel meeting

Sher	1998	The risk is in the transfusion, not the donation	Design	Letter
Sherman	1982	Alanine aminotransferase levels among volunteer blood donors: geographic variation and risk factors	Intervention	Did not include MSM as a risk factor
Shev	1995	Risk factor exposure among hepatitis C virus RNA positive Swedish blood donors--the role of parenteral and sexual transmission	Intervention	Did not include MSM as a risk factor
Shinde	2007	A study-screening of blood donors for blood transmissible diseases	Population	Indian blood donors
Silvani	2000	Blood donors with 'medium' or 'minor' risk factors for human immunodeficiency virus infection: are they eligible for donation?	Population	MSM excluded from study population
Skinner	2010	Science should dictate policy	Design	Opinion piece
Slowther	2013	Evidence-Based Policy and Practice Leads to Changes in the Criteria for MSM to Donate Blood	Design	News item
Smith	2011	Blood donation and institutional trust: risk, policy rhetoric, and the men who have sex with men lifetime deferral policy in Canada	Design	Qualitative study
Soldan	1998	Incidence of seroconversion to positivity for hepatitis C antibody in repeat blood donors in England, 1993-5	Intervention	Risk factors not assessed in control group
Soldan	2003	Evaluation of the de-selection of men who have had sex with men from blood donation in England	design	Modelling study
Sookoian	1997	The problem of hepatitis C virus infection in blood donors. Clinico-epidemiological, virological and histological evaluation	Intervention	Did not include MSM as a risk factor
Soresi	1998	Transmission of hepatitis C virus: a study of the main risk factors in a Sicilian population of volunteer blood donors	Intervention	Did not include MSM as a risk factor
Sothern	2011	The blood service should ask donors about practice, not just partners	Design	Opinion piece
Spada	2001	Changing epidemiology of parenterally transmitted viral hepatitis: results from the hepatitis surveillance system in Italy	Design	No control group
Spencer	2006	Marginal recruitment impact of potential alterations to FDA deferral criteria for men who have sex with men	Design	Letter to the editor
Stephenson	2004	HIV risk-reduction in adolescents	Design	Comment
Stigum	2001	Risk behavior in Norwegian blood donors	Outcome	No infections measured
Stramer	2009	Investigation of reported cases of suspected transfusion transmission of HIV	Design	Narrative text
Strasser	1995	Evaluation of blood donors with equivocal hepatitis C serological results	Intervention	Risk factors not assessed in control group
Strauss	1990	Safety of Iowa's blood supply	Design	Narrative text
Strong	2009	Vital Publics of Pure Blood	Design	Narrative review
Stuyver	1996	Hepatitis C virus (HCV) genotype analysis in apparently healthy anti- HCV-positive Parisian blood donors	Intervention	No risk factors studied
Suarez	1994	The prevalence of anti-HCV positivity among blood donors in Asturias. A clinical-epidemiologic study	Intervention	Did not include MSM as a risk factor
Suarez	1994	A comparative analysis of blood donors with antibodies to the hepatitis C virus, positivity for the hepatitis B surface antigen and hypertransaminasemia in Asturias	Intervention	Did not include MSM as a risk factor
Suarez	1994	Positive Hbsag Blood-Donors in Asturias (Spain) - Current Prevalence and Its Significance	Intervention	Did not include MSM as a risk factor
Sugg	1988	Antibodies to hepatitis B core antigen in blood donors and hepatitis non-A, non-B in blood recipients	Intervention	No risk factors studied
Suligoi	2013	Low perception of the risk of HIV infection among repeat and first-time blood donors in italy	Intervention	Risk factors not assessed in control group
Symington	2011	Blood donor ban upheld	Design	Opinion piece
Szmuness	1973	Hepatitis B antigen and antibody in blood donors: an epidemiologic study	No	Intervention
Tabor	1979	Studies of donors who transmit posttransfusion hepatitis	Intervention	No risk factors studied
Tabor	1981	Antibody to hepatitis B core antigen in blood donors with a	Intervention	Risk factors not

		history of hepatitis		assessed in control group
Talib	1993	Blood safety	Design	Narrative text
Tan	1999	Analysis of hepatitis G virus (HGV) RNA, antibody to HGV envelope protein, and risk factors for blood donors coinfected with HGV and hepatitis C virus	Intervention	Unclear if risk factors were measured in the control group; no further information was obtained after contacting the author
Tedder	1980	Contrasting patterns and frequency of antibodies to the surface, core, and e antigens of hepatitis B virus in blood donors and in homosexual patients	Population	Blood donors vs MSM
Tobler	2010	A case-control study of factors associated with resolution of hepatitis C viremia in former blood donors (CME)	Intervention	Did not include MSM as a risk factor
Vamvakas	2009	Why are all men who have had sex with men even once since 1977 indefinitely deferred from donating blood?	Design	Editorial
Vamvakas	2009	Scientific background on the risk engendered by reducing the lifetime blood donation deferral period for men who have sex with men	Design	Narrative review
Vamvakas	2011	Relative risk of reducing the lifetime blood donation deferral for men who have had sex with men versus currently tolerated transfusion risks	Design	Narrative review of modelling studies
van de Laar	2006	Diversity and origin of hepatitis C virus infection among unpaid blood donors in the Netherlands	Design	No control group
Van Den Berg	2010	SANBS's policy on blood donations from men who have sex with men	Design	Letter
van den Burg	1998	Donor selection: the exclusion of high risk donors?	Design	Narrative text
Van der Bij	2006	Surveillance of risk profiles among new and repeat blood donors with transfusion-transmissible infections from 1995 through 2003 in the Netherlands	Intervention	Risk factors not assessed in control group
Vermehren	2012	High Prevalence of Anti-HCV Antibodies in Two Metropolitan Emergency Departments in Germany: A Prospective Screening Analysis of 28,809 Patients	Design	No control group
Vernillo	2010	Blood is a precious resource--does it really matter who donates it?	Design	Comment
Vreeland	2011	Tainted blood policy	Design	Opinion piece
Vrielink	1998	Transfusion-transmissible infections	Design	Narrative text
Vrielink	2004	Risk factors in anti-HCV ELISA positive blood donors	Intervention	Did not include MSM as a risk factor
Wainberg	2010	Reconsidering the lifetime deferral of blood donation by men who have sex with men	Design	Narrative review
Wang	2004	Comparison of demographic and donation profiles and transfusion-transmissible disease markers and risk rates in previously transfused and nontransfused blood donors	Intervention	Did not include MSM as a risk factor
Wang	2012	Men who have sex with men and its relationship with HIV-1 strains prevailing in the paid blood donors from Zhengzhou city, Henan	Population	China
Wang	2012	An analysis of risk factors for HIV infection among chinese blood donors	Population	China
Ward	1987	Risk of human immunodeficiency virus infection from blood donors who later developed the acquired immunodeficiency syndrome	Design	Narrative text
Ward	1988	Epidemiologic characteristics of blood donors with antibody to human immunodeficiency virus	Design	No control group
Ward	1990	The epidemiology of transfusion-associated (TA) HIV and other retroviral infections in the United States	Design	Narrative text
Warriner	2009	We need to see evidence on risks of gay men giving blood... 'Call for end to ban on gay men giving blood' (November 4)	Design	Opinion piece
Warson	1984	The impact of donor selection in avoiding hepatitis virus transmission	Design	Narrative text
Waters	2011	From the heart	Design	Report
Watkins	2011	SaBTO review of blood donor selection criteria related to sexual behaviour	Design	Editorial
Watson	1999	Risk factors for hepatitis C transmission in the Victorian	Design	Survey

		population: a telephone survey		
Weimer	2002	Hepatitis A virus prevalence in plasma donations	Design	Epidemiologic report
Wejstal	1999	Sexual transmission of hepatitis C virus	Design	Narrative text
Wernet	1986	Practical significance of the AIDS problem in blood donation services	Design	Epidemiologic report
Widell	2009	Exposed or not exposed - That is the question: Evidence for resolving and abortive hepatitis C virus infections in blood donors	Design	Editorial
Wilkinson	2009	Characteristics of Post-Donation Information (PDI) donors: Demographics, intervening donations and differences by PDI reason	Design	Survey
Wilson	1984	Acquired immune deficiency syndrome (AIDS)	Design	Narrative text
Wilson	2014	Three decades of MSM donor deferral policies. What have we learned?	Design	Opinion piece
Wong	1999	Characteristics of hepatitis C-positive blood donors in Victoria, Australia	Intervention	Did not include MSM as a risk factor
Woodfield	1986	AIDS and blood transfusion	Design	Narrative text
Xu	2013	An assessment of hepatitis E virus (HEV) in US blood donors and recipients: no detectable HEV RNA in 1939 donors tested and no evidence for HEV transmission to 362 prospectively followed recipients	Intervention	No risk factors studied
Zehender	1996	HTLV infection in ELISA-negative blood donors	Intervention	No risk factors studied
Zou	2004	Patterns of age- and sex-specific prevalence of major blood-borne infections in United States blood donors, 1995 to 2002: American Red Cross blood donor study	Intervention	No risk factors studied
Zou	2006	Prevalence of selected viral infections among blood donors deferred for potential risk to blood safety	Intervention	Did not include MSM as a risk factor
Zufferey	1992	Prevalence of anti-HCV (C100-3) antibodies in 20.373 blood donors	Intervention	Did not include MSM as a risk factor

In case no authors were listed, the Accesion Number in PubMed (PMID), Cinahl (Cinahl Acc Nr) or Web of Science (WOS) was given in order to be able to retrieve the references.
